# Shoulder Anatomy and Normal Variants

**DOI:** 10.5334/jbr-btr.1467

**Published:** 2017-12-16

**Authors:** Redouane Kadi, Annemieke Milants, Maryam Shahabpour

**Affiliations:** 1 Clinique Saint-Jean de Bruxelles, BE; 2 University Hospital Brussels, BE

**Keywords:** shoulder, anatomy, MRI, CT arthrography, MR arthrography, variants, radiography

## Abstract

The shoulder joint is functionally and structurally complex and is composed of bone, hyaline cartilage, labrum, ligaments, capsule, tendons and muscles. It links the trunk to the upper limb and plays an important biomechanical role in daily activities. Indications for imaging of the shoulder have considerably increased in the last few years. The article focuses mainly on Magnetic Resonance Imaging (MRI) as well as MR and CT arthrography, diagnostic procedures of choice for assessment of internal derangement of the shoulder. Bony components, rotator cuff tendons and muscles can be assessed on MR imaging without arthrographic technique, whereas the articular structures (including glenohumeral ligaments, capsulolabral structures and hyaline cartilage) require arthrography for more accurate assessment. Appropriate MR imaging protocols and sequences and applied MR anatomy of the shoulder (including normal variants) are proposed to help assist management and treatment of common shoulder pathologies encountered (such as rotator cuff tears, impingement syndromes, and instability as well as less frequent causes of shoulder pain). The most common variants and pitfalls are related to the anterosuperior aspect of the shoulder joint. Basic anatomy as well as recent findings are developed, including a new description of the attachment of supraspinatus and infraspinatus tendons at the superior aspect of the humerus, the rotator cable and the superior glenohumeral ligament complex.

## Imaging Modalities

Routine radiography, ultrasound, CT and MR imaging (conventional and arthrography) are the main diagnostic modalities used for diagnosis of abnormalities around the shoulder joint.

### Conventional Radiography

**Figure 1:**
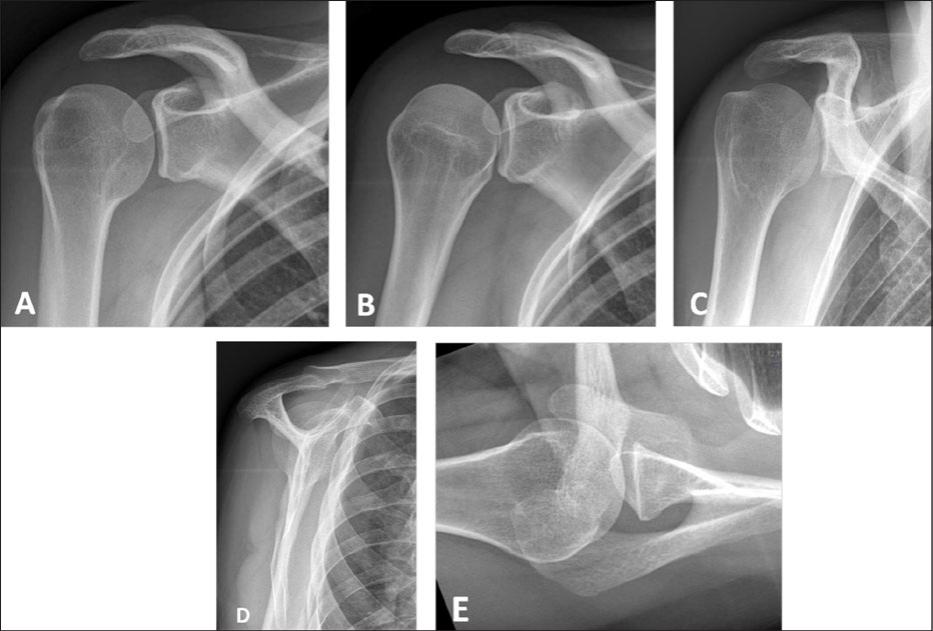
Conventional radiographs of the shoulder. **(A)** Anteroposterior (AP) view with external rotation; **(B)** AP with internal rotation; **(C)** AP with neutral arm position; **(D)** Lateral view of scapula or ‘Y’ view; **(E)** Axillary view.

Although this chapter is based on MRI, we should not forget the importance of standard radiographs for the evaluation of bone and joint structures. Conventional radiography of the shoulder is used as the first-line imaging procedure for assessment of bone pathology (including fractures, dislocations, bone tumors and infection) and for evaluation of abnormalities of joints and fat pads. Limited soft tissue evaluation, ionizing radiation and difficulties of patient positioning (due to pain, fracture, ankylosis etc.) are major limitations. Two orthogonal views (anteroposterior and lateral views) of any bone or joint should be ideally obtained. In daily practice, the anteroposterior view is performed in neutral position and with internal and external rotation of the arm and completed by a lateral view of the scapula (Y view). Additional views with different projections (as an axial view, also called axillary superoinferior view) can be used to explore the shoulder for detection of specific pathologies as described in Table 1 and Figure 1 [1].

**Table 1:** Conventional radiography of the shoulder [1].

Projections	Main visualized anatomy and pathology

Anteroposterior – neutral arm position	Anterior dislocation Fracture of proximal humerus, clavicle, and scapula (i.e. Bankart lesion) Fat-fluid level (erect position)
Anteroposterior – internal arm rotation	Hill-Sachs lesion (posterolateral humeral head impacted fracture)
Anteroposterior – external arm rotation	Trough sign (anteromedial humeral head compression fracture) in posterior dislocation
Scapula ‘Y’ (true lateral view of scapula or outlet view)	Fracture of scapular body, acromion, coracoid process, proximal humerus Humeral head to glenoid fossa relationship
Axillary (superoinferior view)	Humeral head to glenoid relationship
Lawrence (no full abduction required)	Anterior and posterior dislocation
West Point (minimal arm abduction)	Anteroinferior rim of glenoid (West Point view)
Outlet (oblique)	Acromial fracture and morphology Rotator cuff outlet
Grashey (posterior oblique with glenoid in profile)	Glenohumeral joint space (obliterated in posterior dislocation)
Acromioclavicular (without/with stress)	Acromioclavicular joint separation
Bicipital groove (tangent, humeral head)	Bicipital groove
Lateral transthoracic (true lateral view of proximal humerus)	Proximal humeral fracture Humeral head to glenoid relationship

### Ultrasonography

The shoulder joint is well suited to evaluation by ultrasonography (US) because of its easy accessibility. The clinical indications for shoulder US include rotator cuff disorders, bursitis and shoulder impingement. However, ultrasonographic evaluation of the shoulder is limited to the long head of biceps tendon, the rotator cuff, the subacromial-subdeltoid bursa and the acromioclavicular joint. A paramount advantage of US is the dynamic evaluation of shoulder impingement (i.e. in internal & external rotation) [1].

### Computed Tomography

**Figure 2:**
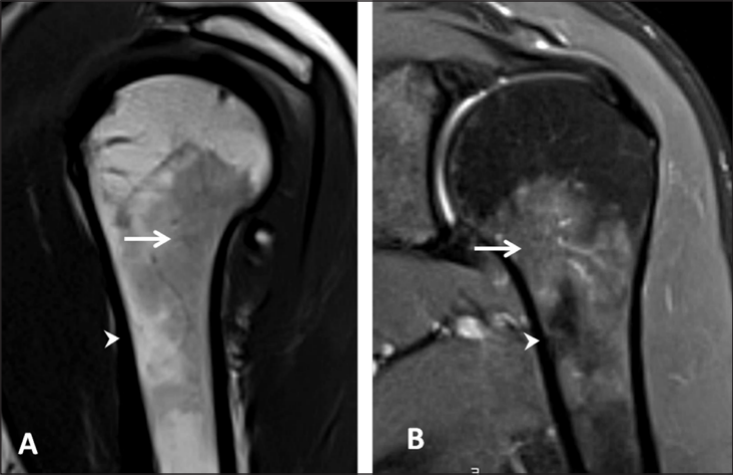
Normal red bone marrow in a young adult. **(A)** Sagittal oblique T1-weighted and **(B)** Coronal oblique fat-suppressed PD-weighted MR images detect areas of red marrow in the proximal humeral diaphysis with low signal intensity on T1 (arrow, A) and increased signal on fat-suppressed PD (arrow, B). This is usually observed bilaterally and without epiphyseal involvement. Cortical bone has a low signal intensity on both sequences (arrowhead, A and B).

Pathologies that are poorly visualized on conventional radiographs are better evaluated with computed tomography (CT). This method provides multiplanar reconstructions, surface rendering of the osseous structures with rotation of the reconstructions and subtraction Figure 2. Complex fractures and dislocations, bony fragments and calcifications as well as the degree of fracture healing are better assessed on CT. In bone tumors or some soft tissue tumors, the matrix calcification (osteoid or cartilaginous) could be precised.

### CT Arthrography

CT arthrography (CTA) is indicated for detection of labral tears and articular cartilage lesions with a higher resolution than MRI. Intra-articular injection of iodine contrast material allows visualization of the capsulolabral structures. The soft tissues are poorly visualized compared to MRI. Other disadvantages include ionizing radiation, invasiveness of injection procedure and the presence of metal artifacts in postoperative patients.

### Magnetic Resonance Imaging

Conventional Magnetic Resonance Imaging (MRI) allows direct evaluation of rotator cuff muscles and tendons, medullary bone and neurovascular structures. The disadvantages include longer examination time and higher cost, respiratory motion artifacts and patient claustrophia. MR images are obtained with a dedicated shoulder coil at 1.5 or 3 Tesla. The patient is placed in supine position with the arm in mild external rotation. Coronal oblique images are oriented parallel to the scapula or parallel to the course of the supraspinatus tendon (determined on axial images); sagittal oblique images are oriented perpendicular to the coronal oblique plane, covering the deltoid muscle and the scapula to include rotator cuff muscle bellies; axial images are performed from the acromioclavicular joint to below the axillary pouch. An example of different pulse sequences is represented in Table 2.

**Table 2:** Example of standard MRI protocol of the shoulder (based on the guidelines of the European Society of Skeletal Radiology (ESSR) Sports Subcommittee 2016).

	FOV (max)	Slice (max)	TE	Matrix (min)

Axial fat suppressed (FS) proton density (PD)	16 cm	3 mm	10–40	256 × 256
Coronal oblique FS PD	16 cm	3 mm	10–40	256 × 256
Sagittal oblique FS PD	16 cm	3 mm	10–40	256 × 256
Sagittal oblique T1	16 cm	3 mm	Min	256 × 256
Coronal oblique T2	16 cm	3 mm	80–100	256 × 256
Axial GRE 2D (or 3D) (optional)	16 cm	2 (or 1) mm	3–10	256 × 256

### MR Arthrography

MR Arthrography (MRA) is necessary for an accurate detection of capsulolabral lesions thanks to the distension of the joint capsule. Direct MRA uses intra-articular injection of gadolinium based contrast with the same technical approach as for CTA. Indirect MRA performed after intravenous contrast injection is less invasive and expensive but lacks capsular distension and therefore is less accurate than direct MRA. An example of different MRA pulse sequences is represented in Table 3.

**Table 3:** Example of standard MRA protocol of the shoulder (based on the guidelines of the European Society of Skeletal Radiology (ESSR) Sports Subcommittee 2016).

	FOV (max)	Slice (max)	TE	Matrix (min)

Axial T1	16 cm	3 mm	Min	256 × 256
Axial FS PD	16 cm	3 mm	10–40	256 × 256
Sagittal oblique FS PD	16 cm	3 mm	10–40	256 × 256
Coronal oblique FS T1	16 cm	3 mm	Min	256 × 256
Coronal oblique FS PD	16 cm	3 mm	10–40	256 × 256
ABER FS T1 (optional)	16 cm	3 mm	Min	256 × 256

## Articular Anatomy

The most flexible joint in the entire human body is the shoulder joint; this is due to a synergistic action of four separate articulations: the glenohumeral, acromioclavicular, sternoclavicular, and scapulothoracic joints [2]. In this issue we focus on glenohumeral and acromioclavicular joints.

### Glenohumeral Joint

**Figure 3:**
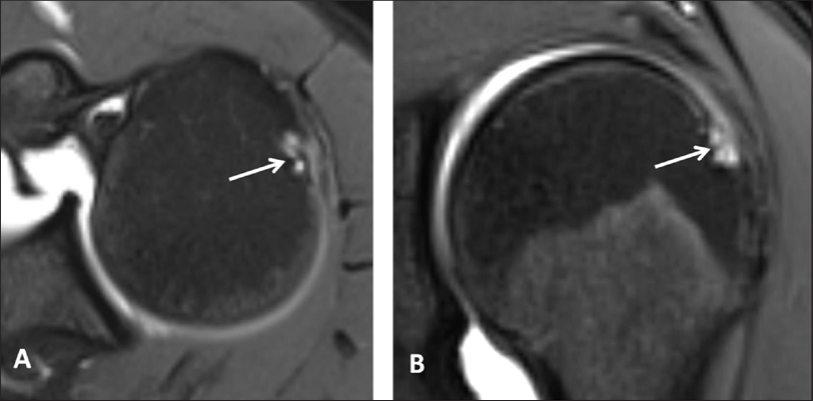
Subchondral cysts of the humeral head and normal bare area. **(A)** Axial and **(B)** Coronal oblique fat-suppressed T1-weighted MR arthrographic images show subchondral cysts at the attachment of the infraspinatus tendon (arrow). Coronal oblique section of the same patient discloses a normal bare area in the posterolateral aspect of the humeral head with small fibrocystic changes that communicate with the joint and should not be mistaken for a cartilage defect (arrow, B). Such changes are common and often asymptomatic.

The glenohumeral joint is a ball-and-socket joint lying between the articulation of the rounded head of the humerus and the cup-like depression of the scapula, also called the glenoid fossa (Figures 1–3, additional material). The glenoid fossa forms a very shallow socket reinforced by muscles, ligaments and cartilage, helping to prevent dislocation. Labrum surrounds the glenoid fossa to extend the size of the socket while maintaining flexibility. To further reinforce the shoulder, the four muscles of the rotator cuff extend from the scapula and surround the head of the humerus to rotate the arm and prevent dislocation. The shoulder is capable of flexion-extension, abduction-adduction, circumduction and medial and lateral rotation. The shoulder anatomy provides mobility but leads to a relatively unstable joint, prone to subluxation and dislocation [2].

### Acromioclavicular Joint

The acromioclavicular joint is formed by an articulation between the lateral end of the clavicle and the acromion of the scapula (Figures 1–3, additional material). It is a flat, gliding joint that gives the shoulder additional flexibility which is not possible with the glenohumeral joint alone. The articular surfaces of the acromioclavicular joint are covered with hyaline cartilage and in the central portion of the joint there is a fibrocartilaginous disc, usually incomplete. The fibrous capsule surrounds the articular margins and is reinforced by the superior and inferior acromioclavicular ligaments and by the coracoclavicular ligaments. The inferior portion of the joint is also reinforced by fibers of the coracoacromial ligament, which blends with the undersurface of the capsule [2].

## Osseous Anatomy

### Proximal Humerus

The proximal humerus consists of the humeral head, the greater and lesser tuberosities, the humeral neck, and the bicipital groove (Figures 1 and 2, additional material). The greater tuberosity is located on the lateral aspect of the proximal humerus and is the site of insertion of the supraspinatus, infraspinatus, and teres minor tendons. The lesser tuberosity is situated on the anterior portion of the proximal humerus, medial to the greater tuberosity. The subscapularis tendon inserts here in a broad band. The anatomic neck forms the oblique circumference of the humeral head and separates the head from the tuberosities. The surgical neck forms the axial circumference of the humerus immediately inferior to the tuberosities and is often involved in fractures. The groove between the two tuberosities along the anterior surface of the humerus is known as the intertubercular or bicipital groove and supports the long head of the biceps tendon. The width of the medial border and depth of the groove both affect the risk of subluxation of the long head of the biceps tendon [2,3,4].

### Cortical and Trabecular Bone

Cortical bone has low signal intensity due to its high density and slow-moving protons. Trabecular bone has high or intermediate signal intensity on T1-weighted images. The epiphysis shows fatty marrow, whereas the metaphysis and diaphysis show variable hematopoietic marrow, depending on the distribution of fatty to hematopoietic marrow [5]. Small residual islands of red bone marrow or larger areas of bone marrow reconversion can be present in the metaphysodiaphyseal region of the proximal end of humerus and are considered as physiological. They present moderate low signal intensity on T1- and T2-weighted images, higher than the muscle signal and an increased signal on fat saturated T2-weighted images. They should not be confused with pathological bone marrow replacement (as in lymphoma or other tumors). Contrarily to benign hematopoietic marrow hyperplasia, those pathologies are characterized by very low signal intensities on T1-weighted images and an asymmetrical distribution bilaterally with epiphyseal involvement (Figure 2) [6].

Subchondral cysts can be present within the humeral head and are normally found at the insertion of the supraspinatus and infraspinatus tendons. The cysts in these locations do not represent degenerative sequels, whereas cysts located more anteriorly are associated with subscapularis tendon pathology. Cystic lesions in the posterosuperior bare area of the humeral head should not be mistaken for degenerative sequels or vascular channels. Instead, they are typically pseudocysts that communicate with the joint space and represent a normal variant (Figure 3) [4,6].

**Figure 4:**
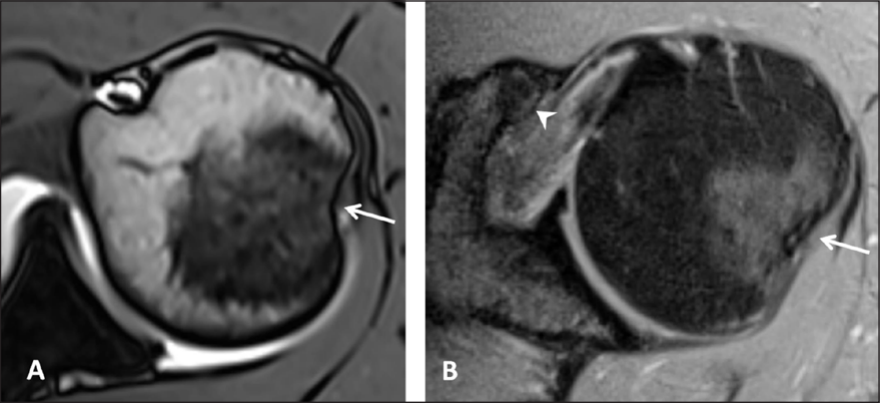
Normal humeral head versus Hill-Sachs lesion. **(A)** On the axial T2 gradient echo weighted MR image, there is a slight flattening of the posteroinferior surface of the humeral head (arrow), which is a normal finding. **(B)** Axial fat saturated T2-weighted MR image obtained at the level of the coracoid process (arrowhead, B) typically shows a Hill-Sachs defect (arrow) in a patient with history of anterior shoulder dislocation.

A variable deep notch or a physiological flattening in the humeral neck is located posterior to the greater tubercle and best visualized on axial images; this pitfall should not be mistaken for a Hill-Sachs impaction which is seen at or above the level of the coracoid process (Figure 4) [4,5]. A normal bare area in the posterolateral aspect of the humeral head, located between the insertion of the posterior capsule and the edge of the articular surface of the humeral head should not be considered as cartilage defect on axial sections. True cartilage defects of the humeral head are often located in the posterosuperior portion medial to the location of the bare area [3,5,6,7].

### Cartilage

The assessment of humeral cartilage remains critical due to the small cartilage thickness at this level (approximately 1mm) [3]. The articular cartilage of the humeral head is thicker centrally and thinner peripherally contrary to the glenoid articular cartilage which is relatively thinner centrally and thicker peripherally [7]. Recognition of normal thinning of peripheral humeral cartilage is essential in order to not mistaken it with posttraumatic or degenerative sequels. Cartilage is better evaluated using CTA than MRA because of the smaller slice thickness of CT images and the clear difference in contrast between the injected high-density contrast material (appearing white) and the grey density of cartilage on CT [4].

### Scapula

The scapula is a triangular bone which consists of the scapular body, the scapular spine, the scapular neck, the acromion, the glenoid fossa and the coracoid process. The dorsal aspect of the scapula is divided by the scapular spine into the supraspinous and infraspinous fossa where the supraspinatus and infraspinatus muscles attach respectively [3,6].

#### Glenoid

The glenoid cavity or fossa forms a glenohumeral joint with the medial aspect of the humeral head (Figures 1 and 3, additional material). The glenoid is pear shaped or oval shaped on sagittal sections (Figure 1, additional material). The glenoid cavity is retroverted, approximately 5° to 7° [8]. Referring to a line connecting the anterior and posterior margins on axial images, three main shapes of the glenoid surface are described: (a) concave, (b) flat or (c) convex [3]. The posteroinferior edge of the glenoid can have various shapes, including normal triangular, rounded or J shaped, and delta shaped (Figure 4, additional material). The two last posterior glenoid rim variants can be associated with varying degrees of posterior shoulder instability due to loss of concavity of the inferior glenoid margin. At the superior aspect of the glenoid, the long head of the biceps attaches to the supraglenoid tubercle [4,6].

**Figure 5:**
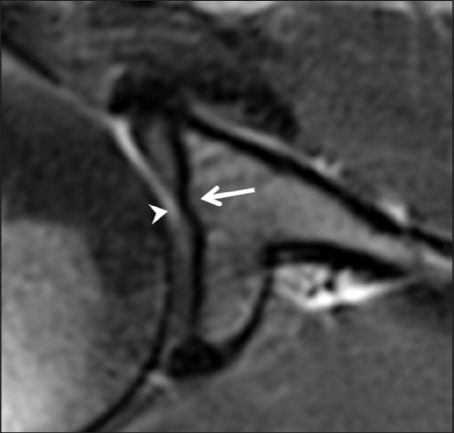
Tubercle of Assaki. Axial fat-saturated PD-weighted MR image shows focal elevation of the subchondral bone (arrow) in the mid third of the glenoid with focal thinning of overlying cartilage (arrowhead).

**Figure 6:**
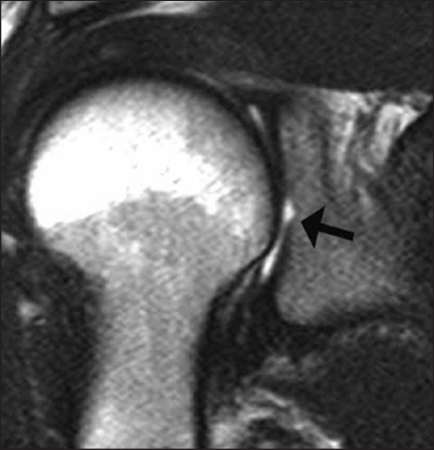
Bare area of the glenoid on MRI. Coronal oblique PD-weighted MR image displays a defect in the cartilage filling up with a moderate amount of joint fluid (arrow) without any thickening of the subchondral bone. As for the tubercle of Assaki, the bare area of the glenoid may be mistaken for a cartilage ulceration.

**Figure 7:**
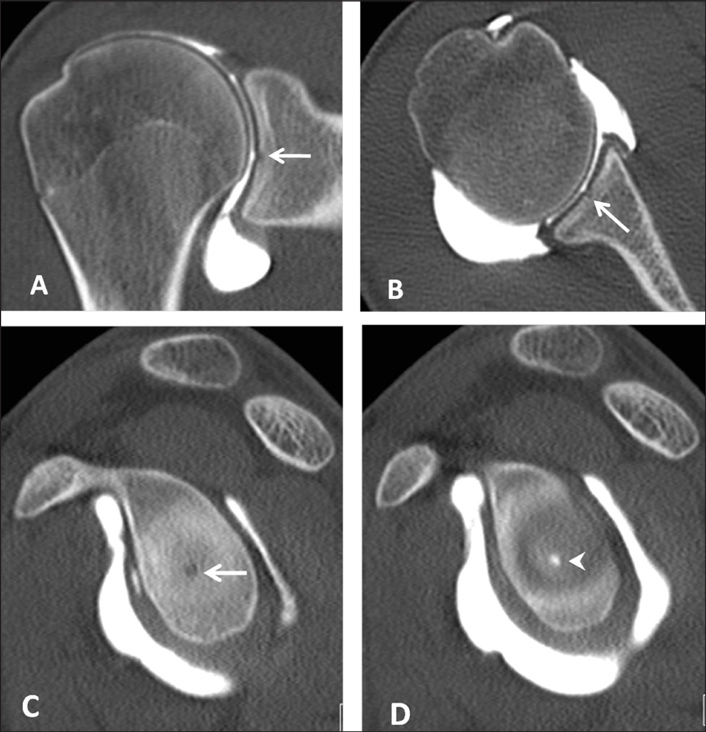
Bare area of the glenoid on CTA. **(A)** Coronal, **(B)** Axial and **(C, D)** Sagittal reconstructed CTA images demonstrate in the mid third of the glenoid a defect of the hyaline cartilage located centrally (arrows, A–C). On D, the defect is filled up by the injected contrast (arrowhead).

The tubercle of Assaki is a ridge (focal zone of elevation) at the subchondral bone in the center of the glenoid cavity (Figure 5). It is associated with a focal thinning of the overlying cartilage. This should not be mistaken for a cartilage defect [3,4]. A bare area has also been described in the mid third of the glenoid cavity; this is an oval area denuded of cartilage, probably developmental and should be differentiated from true cartilage injury (Figures 6 and 7) [6,9].

Glenoid dysplasia is an important developmental abnormality. It comprises an osseous hypoplasia of the posteroinferior glenoid edge in the form of sloping and flattening and is associated with hypertrophy of the adjacent cartilage and labrum and with glenoid irregularity. On axial images a marked retroversion is found. This morphological abnormality may lead to shoulder instability, accelerated osteoarthritis or posterior labral tears [3,6].

#### Acromion

**Figure 8:**
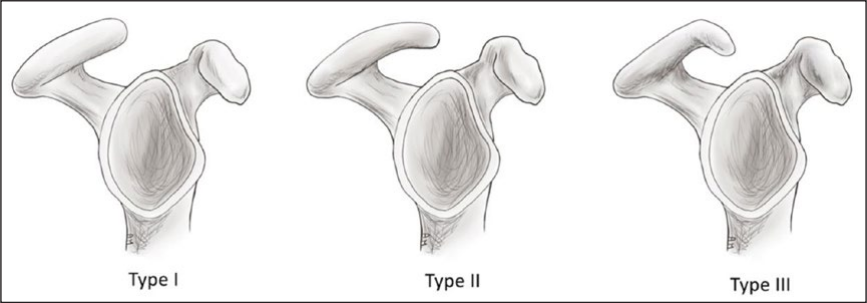
Schematic illustration of the acromion shape as described by Bigliani. **Type I**: flat; **Type II**: curved; **Type III**: hooked.

The acromion is a posterior shoulder landmark; it is a posterolateral extension of the scapular spine, superior to the glenoid. It articulates with the clavicle and is the origin of the deltoid and trapezius muscles. According to Bigliani et al., the acromion is classified into three types: I (flat), II (curved), and III (hooked) (Figure 8). It is hypothesized that the hooked acromion is in fact an acquired form and is highly associated with subacromial impingement syndrome and rotator cuff abnormalities [2,3,4,6,10]. The shape and slope of the acromion is best seen on sagittal oblique sections. On coronal oblique planes the relative location of the acromion to the distal clavicle can be better evaluated. An inferior location of the anterior acromion relative to the undersurface of the distal clavicle has been described as ‘low lying acromion’. An acromion with small slope angle has been described as ‘flat or downsloping acromion’ [5].

**Figure 9:**
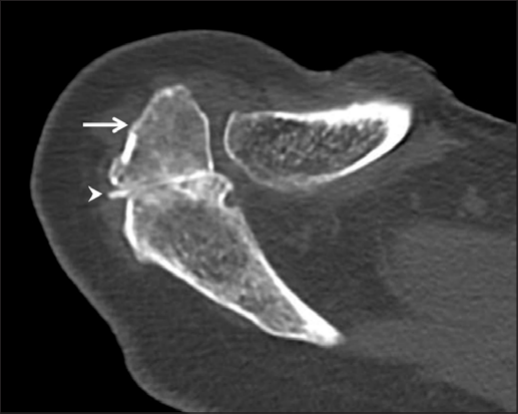
Os acromiale. Axial CT arthrography through the acromioclavicular joint demonstrates an os acromiale (arrow) with synchondrosis (arrowhead). An os acromiale should not be confused with a fracture fragment and the synchondrosis with a pseudarthrosis. In this case, there is a communication between the synchondrosis and the glenohumeral joint (with high-density contrast penetration), which is not normal. Subchondral cystic changes are also seen in the zone of pseudarthrosis.

The os acromiale is an accessory bone due to nonunion of ossification center during development (Figure 9). The incidence of this variant can reach up to 15% of the population [3]. An association between prior shoulder trauma or stress and development of an os acromiale has been reported. It should not be confused with a fracture fragment. Although often asymptomatic, an os acromiale may contribute to clinical symptoms of impingement and might be painful due to mechanical instability and pseudarthrosis formation. Both instability and pseudarthrosis can increase after acromioplasty [4,5,7].

**Figure 10:**
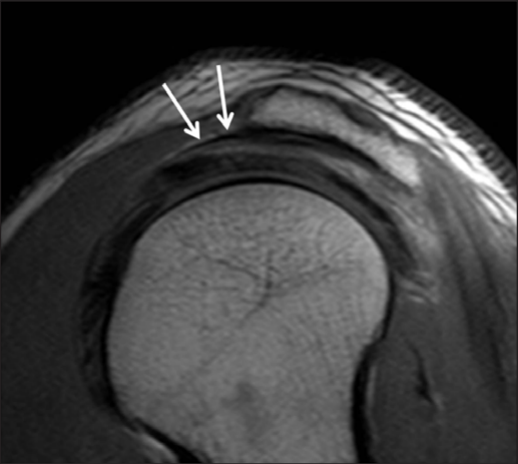
Subacromial pseudospur. Sagittal oblique PD-weighted MR image demonstrates the normal coracoacromial ligament at its acromial attachment that may mimic an osteophyte (arrows).

**Figure 11:**
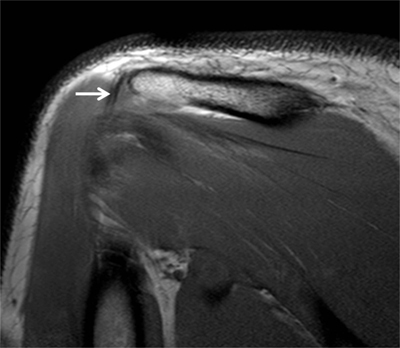
Subacromial pseudospur. Coronal oblique PD-weighted MR image depicts the normal attachment of the tendon of the deltoid muscle visible on one single section mimicking an enthesophyte (arrow). Analysis of consecutive coronal oblique MR images is necessary to avoid misinterpretation.

The subacromial pseudospur is a normal variant that represents a prominence of the acromial angle at the attachment of the coracoacromial ligament. It can mimic an osteophyte caudally directed (Figure 10). Another subacromial pseudospur located at the deltoid tendon attachment to the undersurface of the acromion may mimic an enthesophyte when it is only visible on one single section (Figure 11) [3,4].

#### Coracoid Process

The coracoid process is a hook-shaped bone structure projecting anterolaterally from the superior aspect of the scapular neck, superior and medial to the glenoid fossa. It also represents the tendinous origin of a number of upper extremity and chest wall muscles, including the pectoralis minor and the long head of the biceps brachii. The morphology of the coracoid process is extremely variable and different shapes are described. The coracoid overlap and retroversion angle may have a clinical impact on the subcoracoid space and lead to subcoracoid impingement of the subscapularis tendon between the lesser tuberosity and the coracoid process [3,4,6,11].

### Clavicle

The clavicle is an S-shaped bone which articulates medially with the sternoclavicular joint and laterally with the acromioclavicular joint. It shows many variations from extreme curvature to almost straight shape; increased thickness and curvature can be seen in manual workers [3,4,6].

The different anatomical pitfalls mimicking pathologies are represented in Table 4.

**Table 4:** Normal anatomic structures that may mimic pathology.

Normal variants/Pitfalls	Mimicked Pathologies

Bone marrow reconversion	Pathological bone marrow replacement as in lymphoma or other tumors
Os acromiale	Fracture fragment of the distal acromion or normal acromioclavicular joint
Acromial insertion of the coracoacromial ligament	Acromial pseudospur
Acromial attachment of the deltoid tendon	Acromial pseudospur
Physiological posterolateral flattening of the humeral neck	Hill Sachs lesion
Physiological bare area in the posterolateral aspect of the humeral head	Humeral cartilage defect
Cystic changes of the humeral head	Reactive subchondral cysts of the lesser tuberosity and anterior aspect of the greater tuberosity related to rotator cuff tendinopathy and tears
Cartilage thinning at the tubercle of Assaki of the glenoid	Glenoid cartilage defect
Sublabral recess	Superior labral with anterior and posterior extension (SLAP) tear
Sublabral foramen	Anterior labral tear
Buford complex with an absent anterior superior labrum	Anterior superior labral tear or a displaced labral fragment due to middle glenohumeral ligament attachement directly on the anterosuperior glenoid
Supraspinatus-infraspinatus interdigitation	Tendinopathy
Rotator cable	Rotator cuff tear
Inferior glenohumeral ligament	Displaced labral fragment
Prominent synovial folds of the axillary recess	Loose bodies
Accessory head of the biceps muscle	Longitudinal split tear of the long head of the biceps tendon

## Soft Tissue Structures

To move and support the shoulder, different structures must work in synergy like muscles, tendons, ligaments, and cartilaginous structures. Precise knowledge of the normal anatomy and variants is important to recognize and to identify pathologies.

## Glenoid Labrum

### Normal Labrum

**Figure 12:**
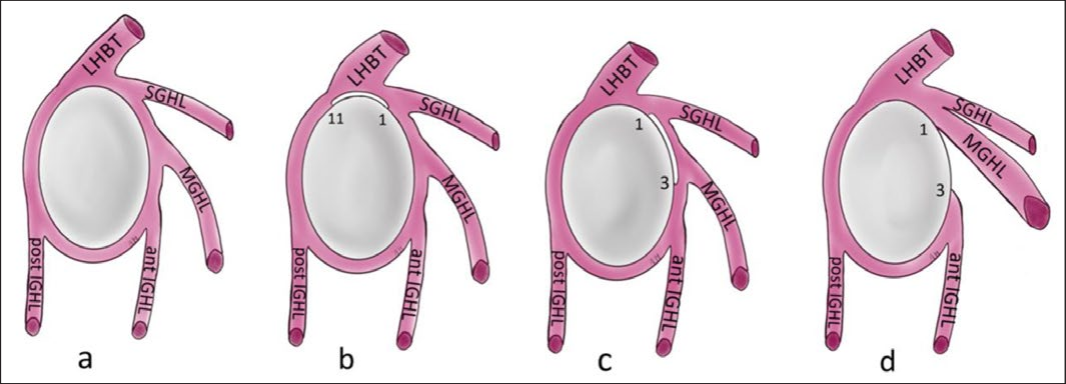
Schematic illustration of the normal capsulolabral complex and anatomical variations. They are shown on a lateral view onto the glenoid. **(a)** Normal anatomy; **(b)** Sublabral recess (sublabral sulcus); **(c)** Sublabral foramen (sublabral hole); **(d)** Buford complex. LHBT: long head of biceps tendon, SGHL: superior glenohumeral ligament, MGHL: middle glenohumeral ligament, IGHL: inferior glenohumeral ligament.

The glenoid labrum is a fibrocartilaginous structure attached around the margin of the glenoid cavity and covering the bony surface. It provides stability of the glenohumeral joint, restricting anterior and posterior displacement of the humeral head. The labrum is larger on the superior aspect than inferiorly. The superior portion of the labrum is closely associated with the biceps tendon (Figure 12). There is variability in size, thickness and morphology of the labrum. Different variations in shape are described anteriorly and posteriorly, as triangular (most common), round, cleaved, notched, flat as well as an absent labrum [2,3,4].

### Labral Variants

#### Sublabral Recess

**Figure 13:**
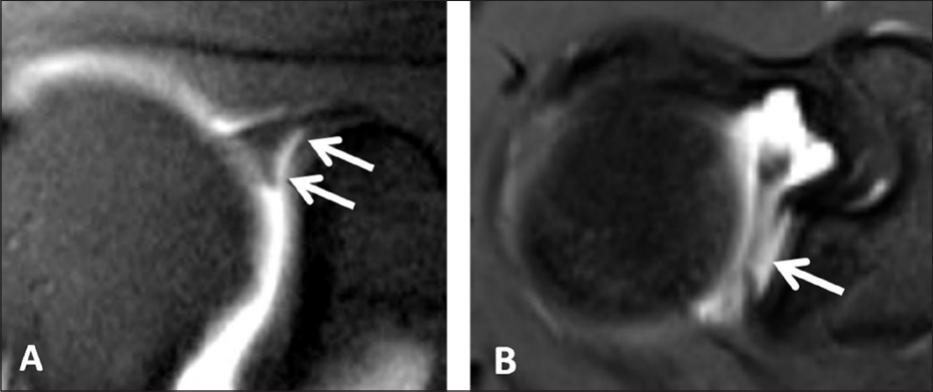
Sublabral recess (sublabral sulcus). **(A)** Coronal oblique fat-suppressed T1-weighted MR arthrographic image shows a sublabral recess as an increased linear signal undercutting the contour of the superior glenoid labrum (arrows, A) following the contour of the glenoid cartilage without extension posterior to the biceps anchor. This sulcus is visualized on **(B)** Axial T1-weighted MR arthrographic image (arrow, B) on an upper section.

Also known as ‘sublabral sulcus’, this physiological recess is most common and represents a variation of the configuration of the biceps labral complex at the 11 and 1 o’clock positions. It presents smooth margins and measures less than 2 mm in width. The sulcus follows the surface of the glenoid rim medially, and does not extend posterior to the biceps anchor (Figure 12) [7]. It corresponds to a synovial reflection medial to the superior edge of the glenoid rim at the biceps anchor, showing a normal defect of the attachment of the superior labrum to the superior glenoid cartilage. The sublabral recess is best seen with arthrographic technique. On CTA and MRA using fat-saturated T1-weighted coronal oblique images, it extends medially toward the glenoid (Figure 13). It should not be mistaken with a type II SLAP lesion or Superior Labrum Anterior Posterior tear which extends laterally or posteriorly [3,4,6,12].

#### Sublabral Foramen

**Figure 14:**
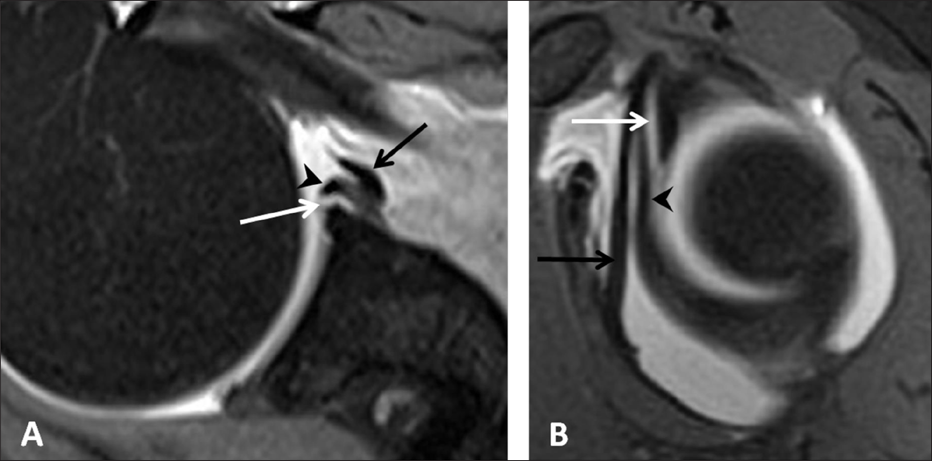
Sublabral foramen (sublabral hole). **(A)** Axial and **(B)** Sagittal fat suppressed T1-weighted MR arthrogram of a sublabral foramen. Sublabral foramen is located between the one o’clock and three o’clock position and provides a communication between the glenohumeral joint and the subscapularis recess (white arrows). Note the smoothly contoured, otherwise normal appearing anterior superior labrum (arrowheads), and middle glenohumeral ligament (black arrows) (Courtesy of Dr Deepu Alex Thomas).

Also known as ‘sublabral hole’, this foramen is less common and represents a normal detachment of the anterosuperior labrum from the underlying glenoid rim at the one and three o’clock positions anterior to the attachment of the biceps labral complex. It presents smooth edges and measures less than 1.5 mm in width. It is oriented medially and posteriorly towards the glenoid (Figure 12). The sublabral foramen provides a communication between the glenohumeral joint and the subscapularis recess [7]. This variant is encountered in about 11% of individuals and best seen on fat-saturated T1-weighted coronal oblique images obtained with MRA and CTA (Figure 14) [13]. The sublabral foramen should not be confused with an anterosuperior labral tear in patients with clinical symptoms. A true tear typically propagates a greater distance superiorly into the bicipital anchor or inferiorly into the inferior glenohumeral ligament attachment site [7]. The sublabral recess can coexist and communicate with the sublabral foramen [3,4,6,12].

#### Buford Complex

**Figure 15:**
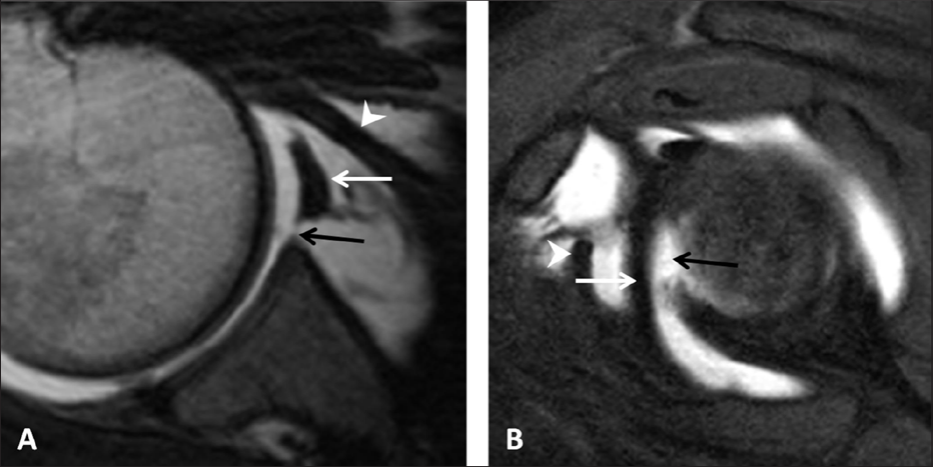
Buford complex. **(A)** Axial PD-weighted and **(B)** Sagittal fat-suppressed T1-weighted MR arthrographic images show a cord-like middle glenohumeral ligament (white arrow) associated with an absent anterior superior labrum (black arrow) mimicking a labral tear with normal posterior labrum. Subscapularis tendon (arrowhead). (Courtesy of Dr Henri Guerini).

The Buford complex represents a combination of two variants which are a significant thickening of the middle glenohumeral ligament with a cord-like appearance and an associated congenital absence of the anterosuperior labrum (Figure 12). This variant is very uncommon and can be encountered in 1.5–2% of individuals [3,6,13]. The thickened middle glenohumeral ligament attaches directly on the anterosuperior glenoid and may be mistaken for a displaced labral fragment [12]. MRA using fat-saturated T1-weighted images and CTA in the axial plane show a cord-like middle glenohumeral ligament adjacent to an absent anterosuperior labrum. Sagittal oblique images could demonstrate the thickened middle glenohumeral ligament and a normal appearance of the superior and inferior glenohumeral ligaments (Figure 15) [4].

## Ligaments

### Glenohumeral Ligaments

**Figure 16:**
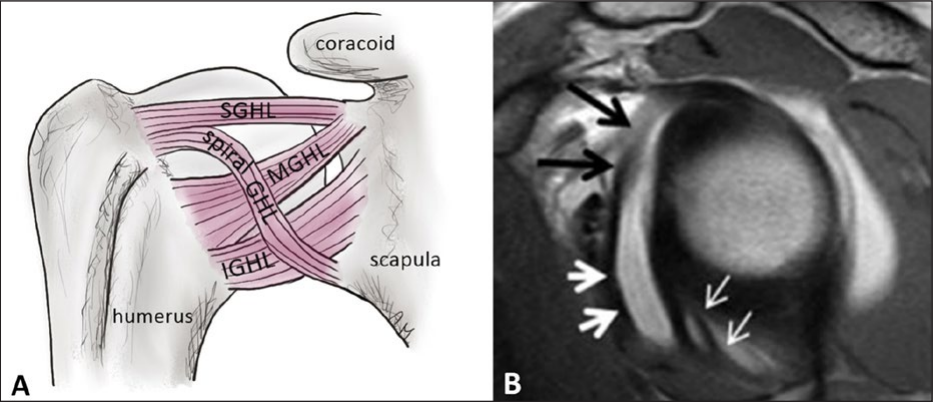
Glenohumeral ligaments and spiral glenohumeral ligament (fasciculus obliquus). **(A)** Schematic illustration of the anterior ligaments of the shoulder. SGHL: superior glenohumeral ligament, MGHL: middle glenohumeral ligament, IGHL: anterior band of the inferior glenohumeral ligament, spiral GHL: spiral glenohumeral ligament or fasciculus obliquus. **(B)** Sagittal oblique PD-weighted MR arthrogram image shows the fasciculus obliquus (thick white arrows, B), the frenula capsulae (synovial bands) (thin white arrows, B) and the middle glenohumeral ligament (black arrows, B) can be identified on this sagittal section.

The glenohumeral ligaments are fibrous reinforcements of the glenohumeral capsule and represent the most important passive stabilizers of the shoulder joint (Figure 12). Classically, three ligaments are recognized: the superior glenohumeral ligament, the middle glenohumeral ligament and the inferior glenohumeral ligament (Figures 12 and 16). They require arthrographic technique (CTA and MRA) for more accurate assessment. They are also easily identified when an articular effusion is present [2,12]. Recent work has expanded their anatomic description for the inferior but also superior glenohumeral ligament complexes. The inferior glenohumeral ligament actually consists of an anterior and posterior band as well as the axillary pouch that is reinforced by the fasciculus obliquus (or spiral glenohumeral ligament) on the glenoid side (Figure 16). Likewise, the superior capsule not only contains the superior glenohumeral ligament, the coracohumeral ligament, and the rotator cable but also the posterosuperior glenohumeral ligament as described by Pouliart et al., [14].

#### Superior Glenohumeral Ligament Complex

**Figure 17:**
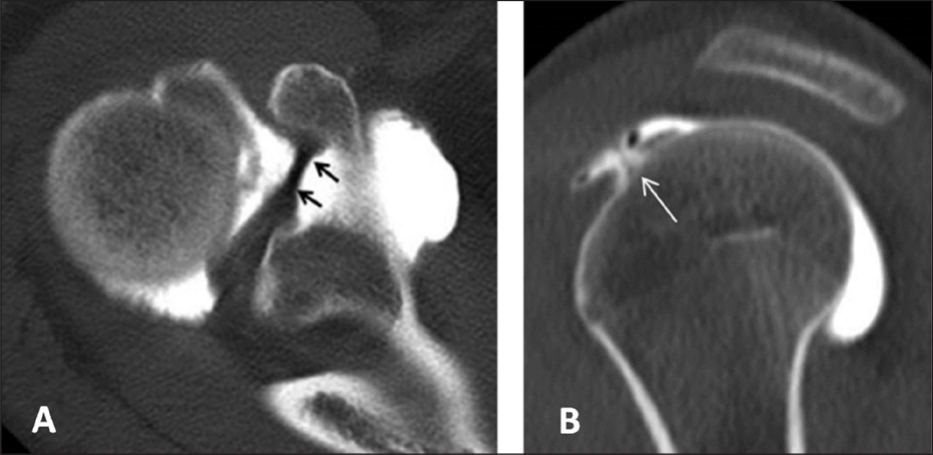
Superior glenohumeral ligament. It can be seen as a medium-size structure running almost straight from the superior labrum into the direction of the coracoid process on axial CTA (black arrows, **(A)**. On sagittal CTA, the ligament appears as a T-shaped structure (thin white arrow, **(B)** Interposed between the long head of the biceps tendon posteriorly and the subscapularis tendon anteriorly.

**Figure 18:**
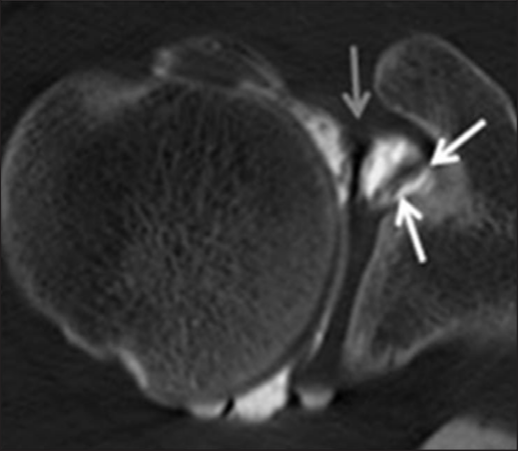
Coracoglenoid ligament is demonstrated on a superior axial CTA image (white arrows). It arises from the supraglenoid tubercle, covering the top of the glenoid rim and superior labrum to insert on the middle of the coracoid process. The superior glenohumeral ligament is indicated by the grey arrow.

**Figure 19:**
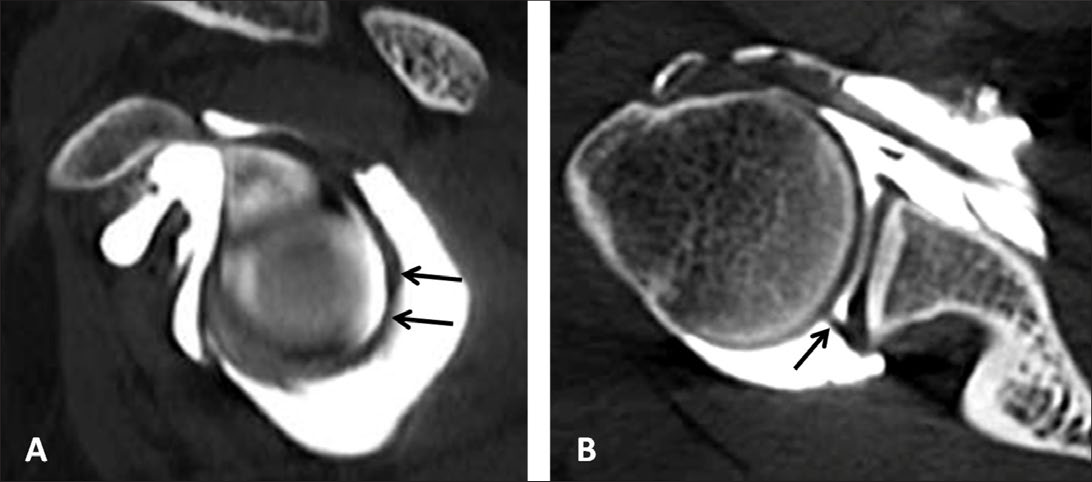
Posterosuperior glenohumeral ligament is demonstrated on **(A)** sagittal and **(B)** Axial CTA images (arrows, A and B). It arises from the posterosuperior part of the glenoid neck, medial to the posterosuperior labrum and the origin of the long tendon of the biceps. Laterally, it fuses with the posterior part of the rotator cable and fibers of the infraspinatus before these three structures jointly insert on the posterior facet of the greater tuberosity.

**Figure 20:**
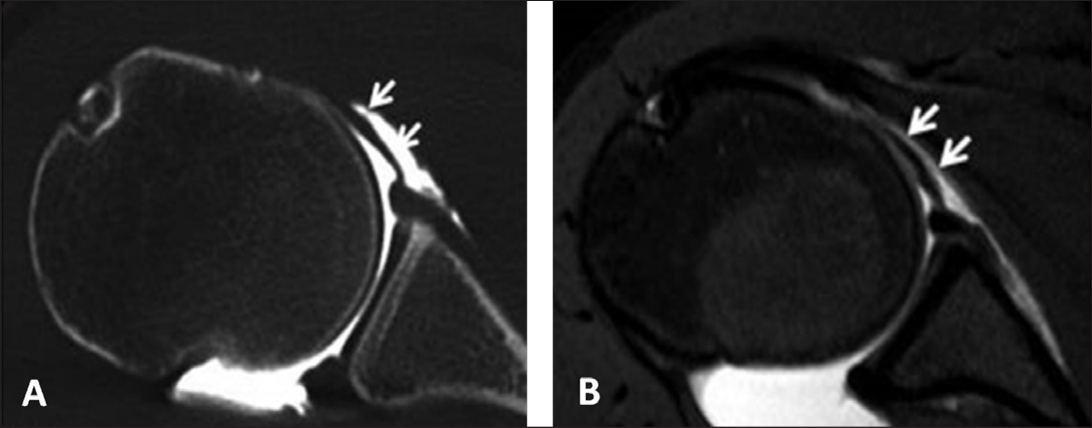
Middle glenohumeral ligament. The midsection of the ligament often adopts a more horizontal course. This can result in a thinner, wavier appearance on axial fat-saturated proton density MRA image (arrows, **B**) with a longer section of the ligament (arrows) on CTA **(A)**.

The superior glenohumeral ligament consists of two proximal attachments, one onto the anterosuperior aspect of the labrum conjoined with the biceps tendon (Figures 12 and 17), and the other onto the base of the coracoid process (Figure 18) [2]. This ligament runs horizontally, almost parallel to the long head of the biceps tendon, straight in the direction of the coracoid process. It courses between the anterosuperior glenoid rim and the humeral head, just above the greater tuberosity (Figure 18) [3]. It forms the limits of the ‘rotator interval’ together with the coracohumeral ligament and the anterosuperior aspect of the glenohumeral joint capsule [4,14]. It can be absent in 10% of healthy subjects [3]. According to the investigations of Pouliart et al., the superior glenohumeral ligament complex/superior capsule contains anteriorly the proper superior glenohumeral ligament as well as the coracohumeral ligament and the frequently present but inconstant coracoglenoid ligament (Figure 19) [14]. A posterosuperior glenohumeral ligament complements the superior glenohumeral ligament complex posteriorly. This ligament originates on the posterosuperior part of the glenoid neck, medial to the labrum and the origin of the biceps tendon. Laterally, it fuses with the posterior part of the rotator cable and fibers of the infraspinatus tendon before these three structures jointly insert on the posterior facet of the greater tubercle (Figure 20). Both anterior and posterior limbs of the superior glenohumeral ligament complex merge with the rotator cable. Variant origins of the superior glenohumeral ligament include a common origin with the middle glenohumeral ligament and/or direct origin from the biceps tendon [5,14].

#### Middle Glenohumeral Ligament

**Figure 21:**
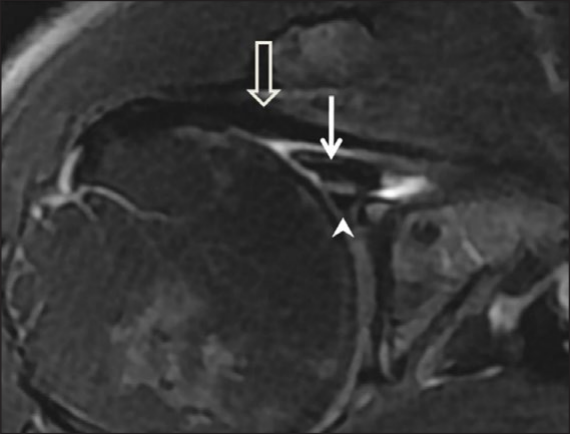
Cord-like middle glenohumeral ligament. Axial fat saturated T2-weighted MR image depicts a thick cord-like middle glenohumeral ligament (arrow). Subscapularis tendon (open arrow) and anterior labrum (arrowhead) are also shown on this section.

**Figure 22:**
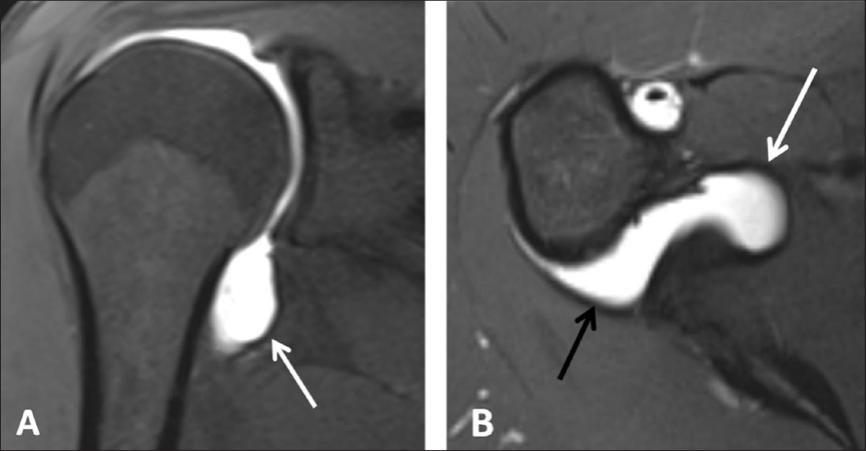
Inferior glenohumeral ligament. On fat-saturated T1-weighted MRA images obtained in **(A)** Coronal oblique and **(B)** Axial planes, the ligament appears as a thin hypointense band delimited by the distended axillary pouch or recess with a U-shaped appearance (arrow, A). The anterior (white arrow, B) and posterior (black arrow, B) bands are demonstrated on the axial section.

The middle glenohumeral ligament originates from the anterosuperior labrum or mid-anterior labrum, in most of cases just below the superior glenohumeral ligament (Figure 12) and runs obliquely to attach to the anatomic neck of the humerus, adjacent to the lesser tuberosity (Figure 21). It can have a conjoined attachment together with the superior glenohumeral ligament, or together with the long head of the biceps tendon when the superior glenohumeral ligament is absent at the 12 and one o’clock positions. The middle part of the ligament lies just posterior to the subscapularis; it may blend together with fibers of the subscapularis muscle. It can vary in size and shape but is usually thin [3,4]. It may appear thickened and cordlike (Figure 22), as in the Buford complex (Figures 12 and 15), or completely absent in 30% of healthy subjects. In that case the capsular recess can be prominent anteriorly and beneath the subscapularis tendon [3,4]. The middle glenohumeral ligament can be doubled as a normal variant. Variant appearances of the middle glenohumeral ligament include absence of the middle glenohumeral ligament, a conjoint origin with either the superior glenohumeral ligament or inferior glenohumeral ligament, and a cord-like thickening of the middle glenohumeral ligament in combination with an absent anterosuperior labrum (Buford complex) [7]. The middle glenohumeral ligament is best visualized on sagittal oblique and axial CT and MR arthrographic images (Figure 20) [4]. The ligament provides stabilization of the glenohumeral joint when the shoulder is abducted 45° [2,6].

#### Inferior Glenohumeral Ligament

**Figure 23:**
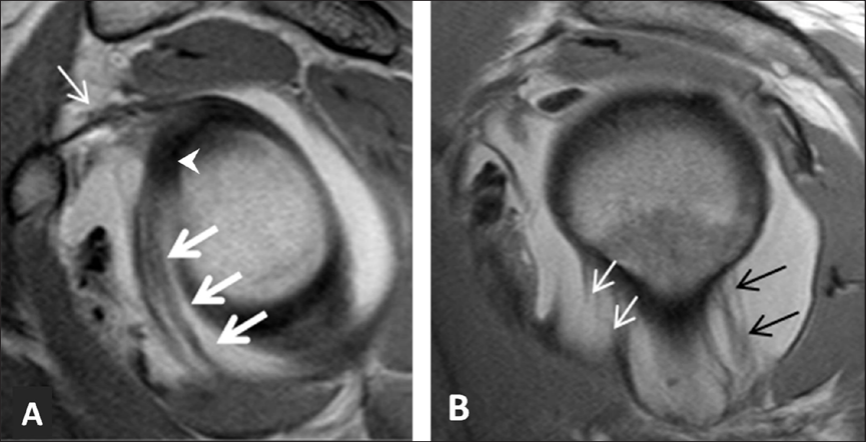
Inferior glenohumeral ligament. **(A)** Sagittal oblique PD-weighted MRA depicts the inferior glenohumeral ligament (thick arrows, A) with a high labral attachment (arrowhead, A). Coracohumeral ligament (thin arrow, A). **(B)** Sagittal oblique PD-weighted MRA shows the anterior band of the inferior glenohumeral ligament (white arrows, B) and the posterior band of this ligament (black arrows, B).

**Figure 24:**
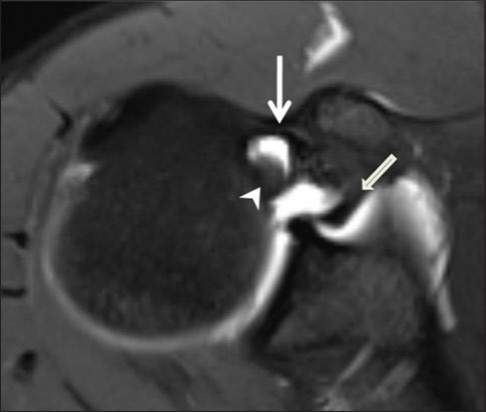
Pulley system. Axial fat-saturated T1-weighted MR arthrographic section at the level of the bicipital groove shows the biceps pulley (large arrow), formed by the fusion of the coracohumeral ligament, the superior glenohumeral ligament (thin arrow) and the transverse humeral ligament. The long head of the biceps tendon is pointed out by an arrowhead.

The inferior glenohumeral ligament is actually a complex of anterior and posterior bands as well as an axillary pouch that is reinforced by the fasciculus obliquus on the glenoid side (Figure 16). The axillary pouch or recess has a U-shaped appearance on MRA or CTA when the inferior glenohumeral ligament is normal (Figures 12 and 23) [4,6,14,15]. As opposed to the other glenohumeral ligaments, its origin is inseparable from the base of the labrum (Figure 12). The anterior band arises from the inferior glenoid rim at the two o’clock to four o’clock positions. The posterior band arises from the inferior glenoid rim at the seven o’clock to nine o’clock position. Both the anterior and posterior bands of the inferior glenohumeral ligament insert along the inferior aspect of the surgical neck of the humerus (Figures 23 and 24) [2,5]. It is the most important glenohumeral ligament in terms of stability; it stabilizes the glenohumeral joint when the arm is abducted to approximately 90° [2,3,6].

### Transverse Humeral Ligament

The long head of biceps tendon is secured within the bicipital groove by the transverse humeral ligament which passes between the greater and lesser tuberosities over the sheath of the tendon. According to the cadaveric study of Gleason et al., there is no identifiable separate transverse humeral ligament. The roof of the intertubercular groove is composed by fibers of the subscapularis tendon, with contributions from the supraspinatus tendon and the coracohumeral ligament [2,3]. The transverse humeral ligament is also intimately related to the biceps pulley (Figure 5, additional material).

### Coracohumeral Ligament

The coracohumeral ligament is not a true ligament connecting two bones. This ligament originates from the coracoid process and terminates on the humeral head where it incorporates into the capsule before attaching on the greater and lesser tuberosities, creating a tunnel for the biceps tendon. As such, it forms the roof of the rotator cuff interval and it covers the anterior aspect of the supraspinatus tendon (Figure 6, additional material). Its posterior attachment to the supraspinatus tendon stabilizes the tendon of the long head of the biceps in the bicipital groove [2,4,6,14]. On axial sections, the coracohumeral ligament is perpendicular to the superior glenohumeral ligament and anterior to the tendon of the long head of the biceps. As described above, the coracohumeral ligament belongs to the anterior limb of the superior glenohumeral ligament complex.

### Coracoglenoid Ligament

As mentioned above, the coracoglenoid ligament belongs to the anterior limb of the superior glenohumeral ligament complex and is recently described as a third ligament in the rotator interval [16]. The coracoglenoid ligament arises from the middle of the coracoid process and inserts posterior to the supraglenoid tubercle, covering the top of the glenoid rim, superior labrum, and long tendon of the biceps. When present, it appears as a straight thin line extending from the superior glenoid rim to the coracoid process on axial and sagittal images (Figure 19) [14].

### Coracoacromial Ligament and Coracoacromial Arch

The coracoacromial arch is an osteoligamentous arch that protects the humeral head and rotator cuff tendons from trauma. It is delimited by the acromion, acromioclavicular joint, coracoid process, and the coracoacromial ligament. It limits the space available to the rotator cuff tendons, the subacromial subdeltoid bursa, and the long head of the biceps (Figure 7, additional material). Compression of either of these structures can lead to subacromial impingement syndrome and/or subacromial bursitis [2].

The coracoacromial ligament is the ligamentous compound of the coracoacromial arch. It is a strong fibrous triangular band that forms part of the roof of the glenohumeral joint. It extends from the edge of the acromion, anterior to the articular surface of the acromioclavicular joint, to the lateral border of the coracoid process (Figure 7, additional material). This ligament is composed of two conjoined or closely adjacent bands [2].

### Coracoclavicular Ligament

The coracoclavicular ligament complex, which connects the distal end of the clavicle to the coracoid process, controls vertical stability of the acromioclavicular joint. It is composed of two separate bundles, the trapezoid and conoid ligaments. The anterolateral trapezoid and posteromedial conoid ligaments are identified on coronal oblique and sagittal oblique sections. The main function of these ligaments is to prevent upward dislocation of the clavicle (Figure 2, additional material) [2,11].

### Acromioclavicular Ligament

The acromioclavicular ligament is divided into superior and inferior part. The superior acromioclavicular ligament extends from the upper acromion to the end of the clavicle. This ligament controls horizontal stability of the acromioclavicular joint. The inferior acromioclavicular ligament is thinner than the superior; it covers the lower part of the joint, and is attached to the two bones along their adjoining surfaces [6].

## Joint Capsule, Recesses and Joint Stabilizers

On MR imaging the normal capsule appears as a low signal line adjacent to the scapular periosteum [14]. It is lined by a synovial membrane [2]. The joint capsule inserts into the glenoid margin of the scapula and the anatomic neck of the humerus. There are two main recesses of the capsule, the subscapular recess and the axillary recess (Figure 23). The *subscapular recess* is located between the coracoid process superiorly and the superior margin of the subscapularis tendon. The *axillary recess* is located between the anterior and posterior bands of the inferior glenohumeral ligament [1]. Prominent synovial folds of the axillary recess may stimulate loose bodies on MRI. The fact that these folds are in the nondependent position of the recess will help distinguish them from true loose bodies [7]. The anterior capsular insertion can be subdivided into three types depending on the proximity of the capsular insertion to the glenoid margin. In *type I*, the capsule appears to attach at the glenoid margin and labrum. In *type II*, the capsule attaches on the glenoid neck within 1 cm of the labral base. In *type III*, the attachment is more than 1 cm medial to the labrum (Figure 8, additional material). Redundancy or type III is commonly observed for the posterior capsule. For the anterior part, this is more variable. Despite the continuity of labrum and most of the capsuloligamentous structures, distension of the joint may also result in the appearance of three distinct types of medial capsular attachment at the inferior attachment [14]. When the anterior capsular attachment is far from the glenoid margin (type III), the glenohumeral joint will be more unstable. However, the appearance of the anterior capsular insertion may vary with the arm in internal or external rotation. In internal rotation, the capsular insertion may appear more medial (type III), and with the arm in external rotation it may appear more lateral (type I) [1]. The capsular mechanism provides the most important contribution to the stabilization of the glenohumeral joint. The anterior capsular mechanism includes the anterior capsule, the glenohumeral ligaments, the synovial membrane and its recesses, the glenoid labrum, the subscapularis muscle and tendon, and the scapular periosteum. The posterior capsular mechanism is formed by the posterior capsule, the synovial membrane, the glenoid labrum and periosteum, as well as the posterosuperior tendinous cuff and associated muscles (supraspinatus, infraspinatus, and teres minor). The long head of the biceps tendon inserting in the superior aspect of the labrum and the triceps tendon inserting on the infraglenoid tubercle inferiorly constitute additional supportive structures of the glenohumeral joint [1].

## Rotator Cuff

The rotator cuff allows the range of motion of the shoulder and protects and stabilizes the glenohumeral joint; it includes the muscles and tendons of the supraspinatus, infraspinatus, subscapularis and teres minor (Figures 2 and 3, additional material). All muscles originate from the scapula. The subscapularis lies anterior to the scapular body, whereas the supraspinatus, infraspinatus, and teres minor lie posteriorly from superior to inferior. The tendons of the cuff insert to the humerus on their respective attachment sites with the subscapularis inserting on the lesser tuberosity and the other three muscles inserting on the greater tuberosity [2,3,4,5]. The supraspinatus muscle arises from the supraspinous fossa along the dorsal scapula. The study of Guerini et al., indicates that the supraspinatus tendon may consist of two distinct strings representing the superficial and deep bundles of the tendon. According to his theory, a full-thickness tear will correspond to a rupture of both bundles, a partial-thickness tear to a rupture of one of the two strings. An interstitial lesion is located between the two strings at the insertion. A cleavage tear is a gap running between the tendon fibers of the two strings (Figure 9, additional material) [18]. The infraspinatus muscle arises from the infraspinous fossa along the dorsal scapula. The supraspinatus and infraspinatus tendons interdigitate and have a partly continuous attachment on the greater tuberosity. The interdigitation is more prominent when the shoulder is internally rotated and should not be confused with tendinopathy on MR imaging [7]. According to the study of Mochizuki et al., the supraspinatus insertion area is smaller and more anterior than suggested in the classic description and the supraspinatus tendon is partially covered by the infraspinatus tendon. The infraspinatus inserts on approximately half of the superior facet and the entire middle facet of the greater tuberosity. The insertion area of the supraspinatus is located at the anteromedial part of the superior facet and is sometimes located at the most superior part of the lesser tuberosity (Figure 10, additional material) [17]. The subscapularis muscle arises from the subscapular fossa of the anterior face of the scapula and attaches to the lesser tuberosity. The supraspinatus and subscapularis tendons interdigitate as well and envelop the biceps tendon. The teres minor muscle arises from the dorsolateral scapula; it inserts into the lowest or most posterior part of the facets of the greater tuberosity. Some fibers of the teres minor interdigitate with those of the infraspinatus [2,3].

Using MR imaging, the rotator cuff is well demonstrated on sagittal oblique T1-weighted sections. The supraspinatus muscle is best demonstrated on coronal oblique and axial sections as a thick, intermediate signal intensity structure tapering into a low signal intensity tendon that inserts into the superolateral aspect of the greater tuberosity. The infraspinatus and teres minor muscles are best demonstrated on axial images as fusiform intermediate signal intensity structures parallel and inferior to the supraspinatus. The subscapularis muscle is located anteriorly and appears on axial sections as an intermediate signal intensity structure coalescing into multiple low signal intensity tendinous portions anteriorly which form one tendon merging with the anterior aspect of the capsule before inserting into the lesser tuberosity [2,3,4,5].

The supraspinatus muscle is required for normal lateral abduction of the upper extremity. The infraspinatus muscle allows external rotation and posterior abduction of the upper extremity. Both supraspinatus and infraspinatus muscles are innervated by the suprascapular nerve. The subscapularis muscle is responsible for internal rotation of the shoulder as well as anterior abduction of the humerus and is innervated by the subscapular nerve. Along with the infraspinatus, the teres minor muscle assists in external rotation of the shoulder and is innervated by the axillary nerve [4].

## Rotator Cable

The rotator cable or ligamentum semicircular humeri is a band-like fibrous thickening that extends in an oblique direction from the coracohumeral ligament along the articular surface of the supraspinatus fibers anteriorly. In its posterior insertion area, the rotator cable is a connecting structure between the teres minor, infraspinatus and supraspinatus tendons (Figure 11, additional material). The rotator cable stabilizes these tendons. The distal fibers of the supraspinatus, infraspinatus and teres minor extending lateral to the rotator cable and inserting into the greater tuberosity of the humerus, are called the ‘rotator crescent’. The connection between the rotator cable and rotator cuff tendons is tight and confirms the ‘suspension bridge theory’ for rotator cuff tears in most areas between the supraspinatus tendon and rotator cable. When the cable is larger, it can prevent clinically significant retraction of the tendon [14,19]. Individuals with a larger cable are termed ‘cable dominant’. Recognition of the cable is important in order to distinguish it from a tear [7]. In a 2013 review paper, the rotator cable was seen in approximately 75% of MRI studies in either the sagittal or the coronal plane, usually 1.3 cm medial to the greater tuberosity enthesis [20]. On arthroscopic images, the rotator cable appears as a fibrous transverse band surrounding the rotator crescent. It can be identified on sagittal and coronal MR arthrographic images as a thin line of intermediate signal intensity interposed between the cartilage of the humeral head and the supraspinatus tendon. (Figure 12, additional material) [14].

## Long Head of Biceps Tendon, Bicipital Anchor and Pulley System

The *long head of the biceps tendon* originates mostly from the supraglenoid tuberosity and partly from the superior labrum, having a common attachment with the superior glenohumeral ligament (Figures 3, 16). This conjoined structure is called the ‘biceps labral complex’ or *bicipital anchor*, where the fibrous tissue of the labrum blends with the biceps tendon (Figure 17). It may originate from the anterior, posterior or both aspects of the labrum. The tendon passes within the joint superiorly and obliquely under the rotator cuff, between the supraspinatus tendon and the subscapularis tendon through the ‘rotator interval’. The long head of biceps tendon is secured within the bicipital groove by the ‘transverse humeral ligament’ which passes between the greater and lesser tuberosities over the sheath of the tendon. The ligament is composed by fibers of the subscapularis tendon, with contributions from the supraspinatus tendon and the coracohumeral ligament [2,3]. The *biceps pulley*, also known as the ‘biceps sling’, is comprised of a combination of the coracohumeral, superior glenohumeral and transverse humeral ligaments. The long head of biceps tendon is covered by a synovial sheath that communicates with the joint capsule. It is best seen on axial images as a circular, signal void structure in the intertubercular groove. The tendon of the short head of the biceps muscle is anterior to the humeral head. Together with the coracobrachialis muscle tendon it originates from the coracoid process and is well demonstrated on axial sections [2,3,4,5,12].

Three types of biceps labral complex (bicipital anchor) have been described. In type 1 the biceps labral complex has a firm attachment to the superior glenoid rim (no sublabral sulcus). Types 2 and 3 are classified according to the varying depth of the sublabral sulcus. Type 2 forms a small sulcus at the superior pole of glenoid. Type 3 corresponds to a large sublabral sulcus which extends under the labrum and over the cartilaginous portion of the glenoid fossa [3]. One of the most common muscular variants is the *accessory head of the biceps muscle*. The supernumerary head is thought to be present in 9.1–22.9% of the population, more commonly seen in Asians. Knowledge of this variant is important not to mistaken it for a longitudinal split tear of the long head of the biceps tendon [4].

## Rotator Interval

The rotator interval contains several important anatomical structures that contribute to the stability and normal function of the shoulder joint, including biceps tendon, coracohumeral ligament, superior glenohumeral ligament, rotator interval capsule, anterior fibers of the supraspinatus tendon, and superior fibers of the subscapularis tendon. It is a triangular area between the anterior border of the supraspinatus tendon and the superior border of the subscapularis tendon, ranging from the coracoid process to the biceps groove. At the capsuloligamentous level, the roof of this space is formed by the anterior part of the superior complex (the superior glenohumeral ligament and coracohumeral ligament). The inferior border of the rotator interval is formed by the middle glenohumeral ligament [6,14].

## Other Muscles

In addition to the principal muscles that act on the glenohumeral joint (rotator cuff and biceps mechanism), other important muscles act on this joint which are briefly summarized: the deltoid muscle originates from the lateral clavicle, acromion, scapular spine and inserts onto the deltoid tuberosity of the humerus. The teres major originates from the inferior lateral scapula and inserts onto the medial intertubercular humeral groove. The trapezius originates from the thoracic spinous processes and inserts into the distal clavicle, acromion and scapular spine. The latissimus dorsi originates from the spinous processes T6–T12 and inserts into the medial intertubercular humeral groove. And the pectoralis major originates from the inferomedial clavicle, sternum and costochondral junctions and inserts into the lateral intertubercular humeral groove. Various muscle variants exist within the shoulder, including accessory biceps brachii muscle heads (described above), coracobrachialis brevis muscle, accessory subscapularis muscle, and the aberrant muscle bundle originating from the latissimus dorsi or pectoralis muscles. Some of those muscle are represented in (Figure 4) [5,6].

## Bursae

There are several bursae around the shoulder, the most important being the subacromial, subdeltoid, subscapular, and subcoracoid bursae (Figure 13, additional material). The subacromial and subdeltoid bursae are sometimes seen as one large continuous bursa called *subacromial subdeltoid bursa*. This bursa is bounded superiorly by the deltoid, acromion, and coracoacromial ligament and inferiorly by the rotator cuff, in particular the supraspinatus. The role of this bursa is to decrease frictional forces on the supraspinatus tendon and between the deltoid and the rotator cuff. Under normal circumstances this bursa does not communicate with the joint space and is not seen on MRI unless it is distended by fluid. However, in the setting of a rotator cuff tear, a communication between the two spaces can develop. The *subcoracoid bursa* is located between the subscapularis muscle and the coracoid process, whereas the superior subscapular recess also known as the *subscapular bursa* is located between the anterior surface of the scapula and the subscapularis muscle (Figure 13, additional material). The subcoracoid bursa does not communicate with the glenohumeral joint and is separated from the subscapular recess by an identifiable fibrous septum; it may communicate with the subacromial subdeltoid bursa in about 10% of patients. The normal subcoracoid bursa is usually not identified on MRI unless distended by fluid. Additional smaller bursae exist within the shoulder and are not commonly visualized on MR imaging. These smaller bursae generally do not communicate with the glenohumeral joint and include the infraspinatus, teres major, and pectoralis major bursae [1,4,5].

## Neurovascular Structures

The suprascapular nerve traverses posteriorly the suprascapular fossa through the suprascapular notch. In this region, the nerve passes beneath the superior transverse scapular ligament (transverse or suprascapular ligament). The suprascapular vessels project superior to this ligament. Just distal to this ligament, the suprascapular nerve sends off several branches. One or two of these branches supply the supraspinatus muscle. The nerve then traverses the spinoglenoid notch to enter the infraspinatus fossa. The inferior transverse scapular ligament (spinoglenoid ligament) forms the roof of the notch. Distal to the spinoglenoid notch, the suprascapular nerve divides in two or more muscular branches that supply the infraspinatus muscle (Figure 14, additional material). Regarding muscle abnormalities as muscle atrophy, involvement of both the supra- and infraspinatus muscles suggests a proximal lesion in the region of the suprascapular notch; involvement of the infraspinatus muscle alone suggests a distal lesion in the region of the spinoglenoid notch [6].

The teres minor and deltoid muscles are innervated by branches of the axillary nerve passing through the quadrilateral (quadrangular) space created between the humeral shaft, the triceps muscle, and the teres major and minor muscles where also passes the posterior humeral circumflex artery. Medial to the triceps muscle is the triangular space, bordered superiorly by the teres minor muscle and inferiorly by the teres major muscle. This space contains the scapular circumflex artery (Figure 3, additional material) [1,2].

## Additional File

The additional file for this article can be found as follows:

10.5334/jbr-btr.1467.s1 Shoulder Anatomy and Normal Variants Including normal MR images in the 3 planes and anatomical drawings and illustrations.
