# Supplementary material for: Shoulder Anatomy and Normal Variants
Source: J Belg Soc Radiol. 2017 Dec 16;101(Suppl 2):3. doi: 10.5334/jbr-btr.1467 (PMC6251069; doi:10.5334/jbr-btr.1467)
Supplement: Shoulder Anatomy and Normal Variants — Including normal MR images in the 3 planes and anatomical drawings and illustrations. [file jbsr-101-2-1467-s1.pdf]

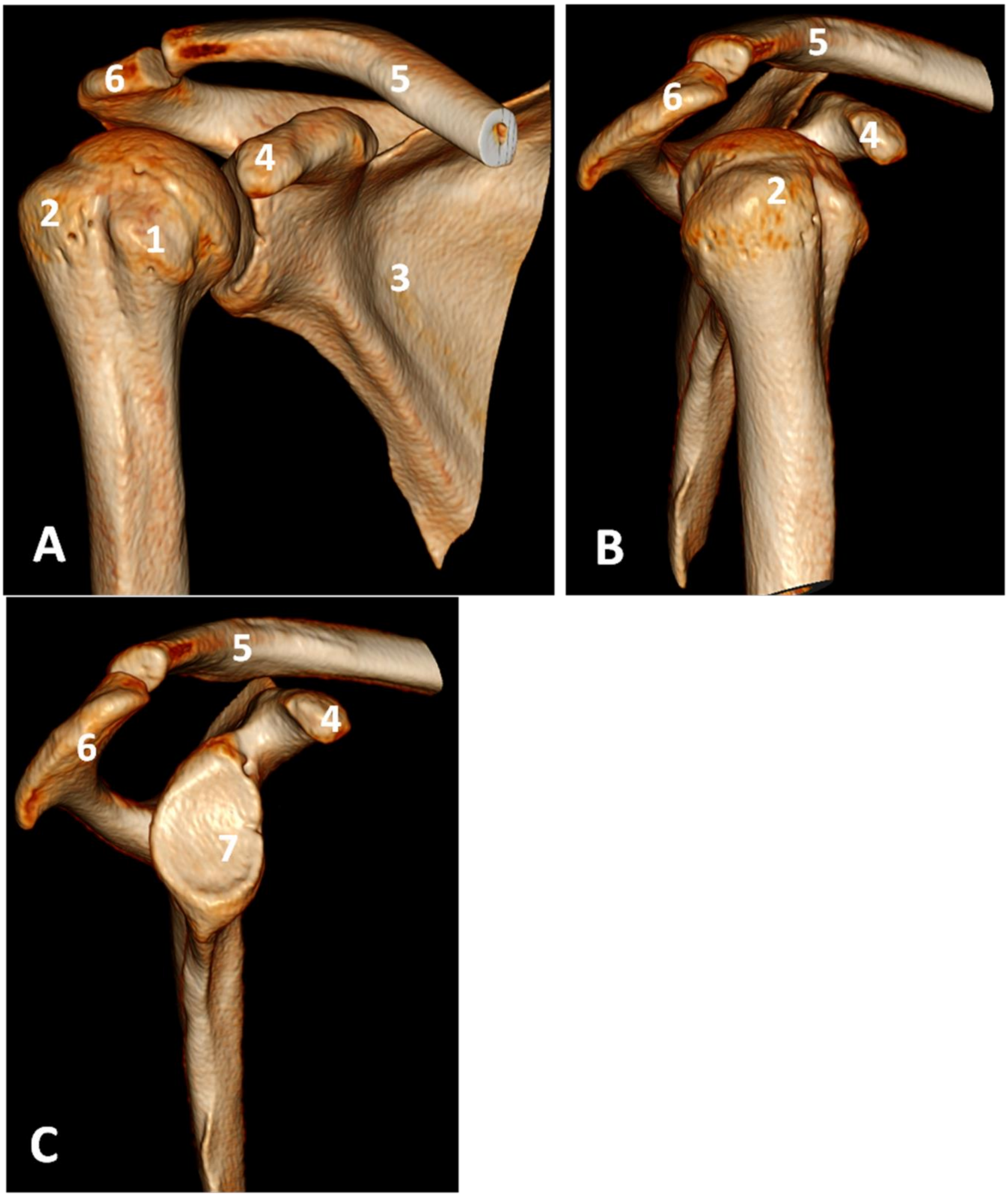

Figure 1, additional material: Volume rendering of the shoulder joint. A: anteroposterior view; B: lateral view; C: glenoid with a characteristic pear shape after subtraction of humeral head. 1: lesser tuberosity; 2: greater tuberosity; 3: scapula; 4: coracoid process; 5: clavicle; 6: acromion; 7: glenoid fossa.

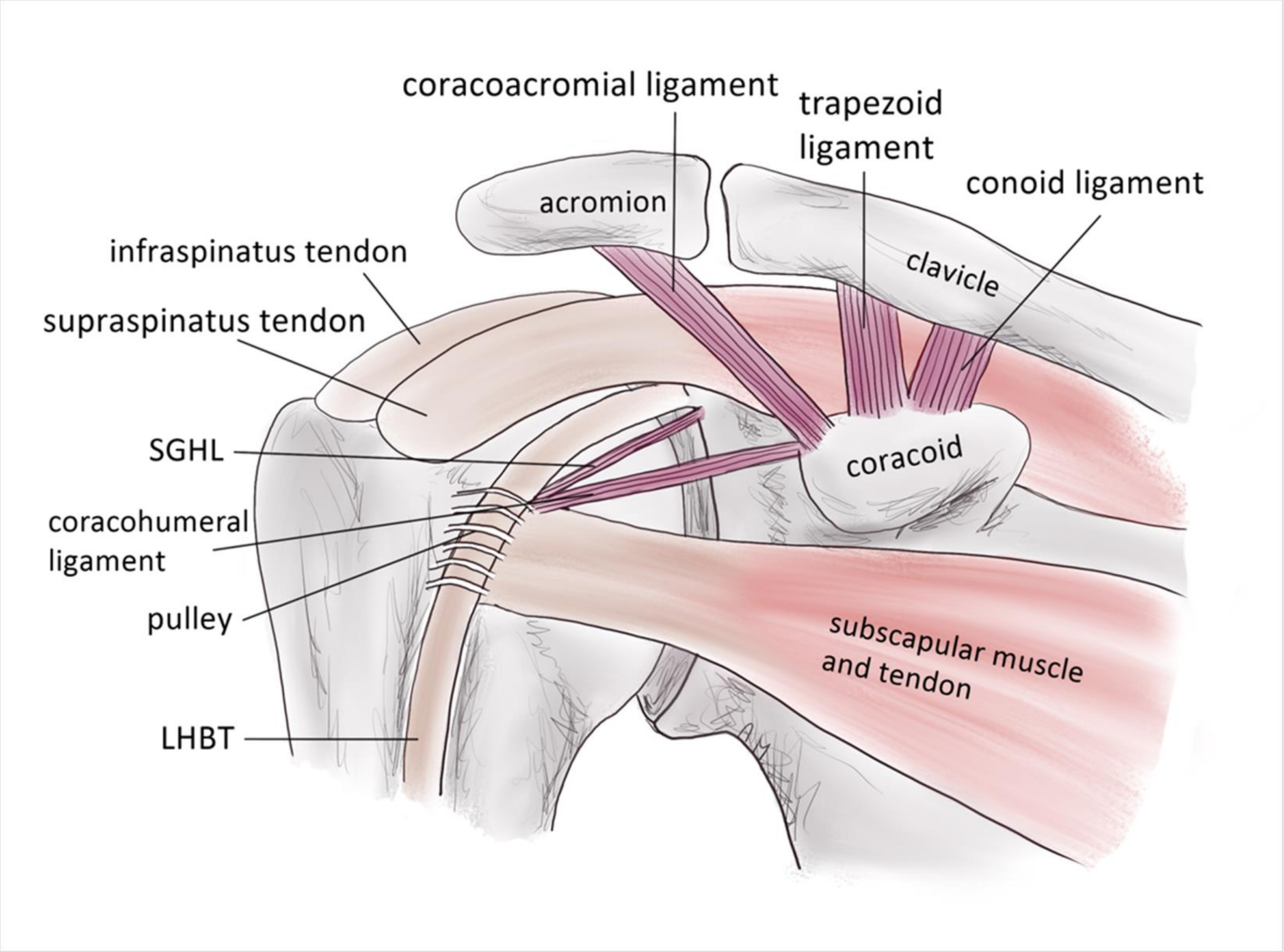

Figure 2, additional material: Schematic illustration of simplified shoulder anatomy. LHBT: Long head of biceps tendon; SGHL: Superior glenohumeral ligament. Trapezoid ligament and conoid ligament forming the coracoclavicular ligament.

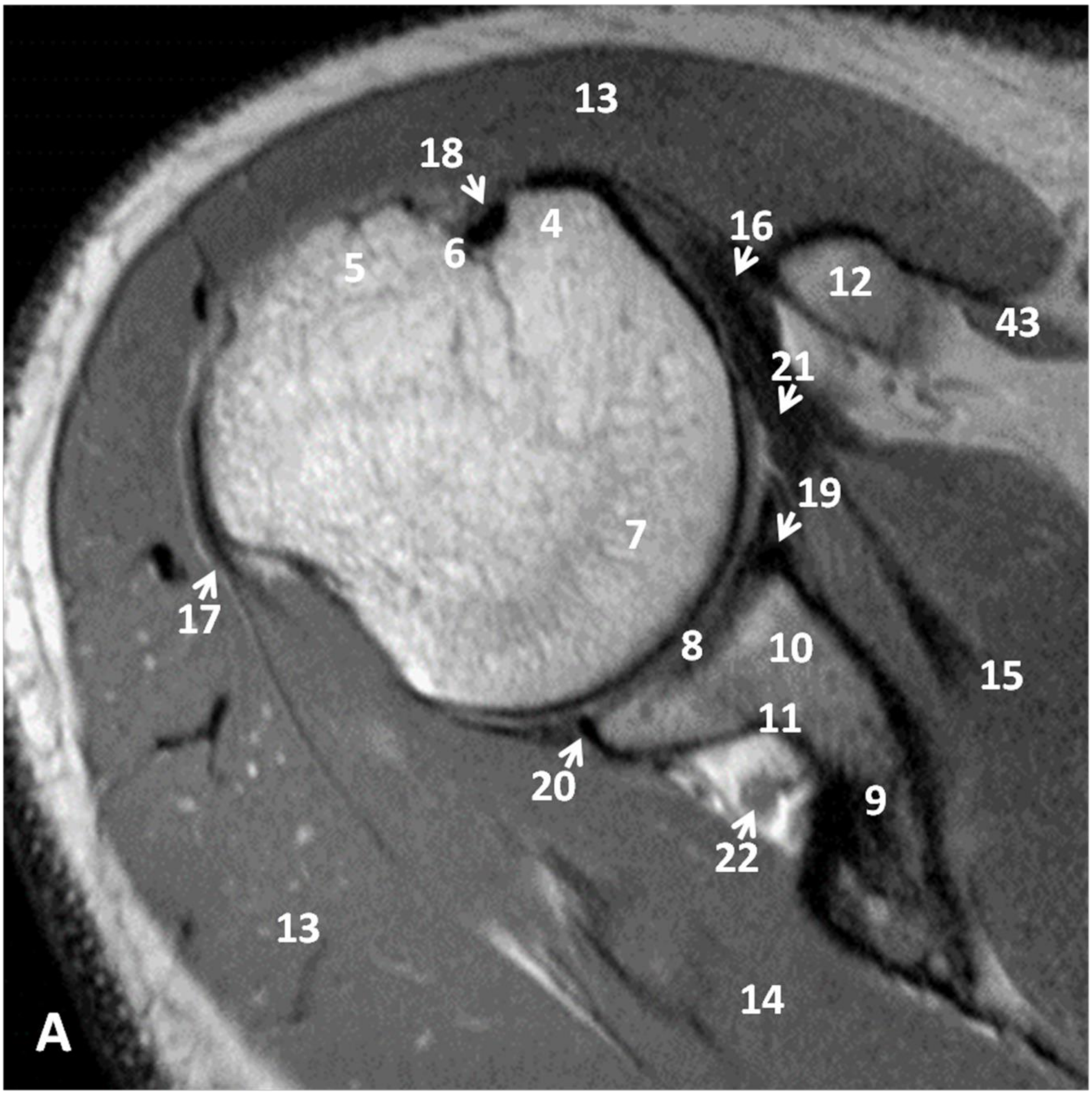

13
18
4
6
5
16
12
43
21
19
7
17
8
10
15
11
20
9
22
13
14
A

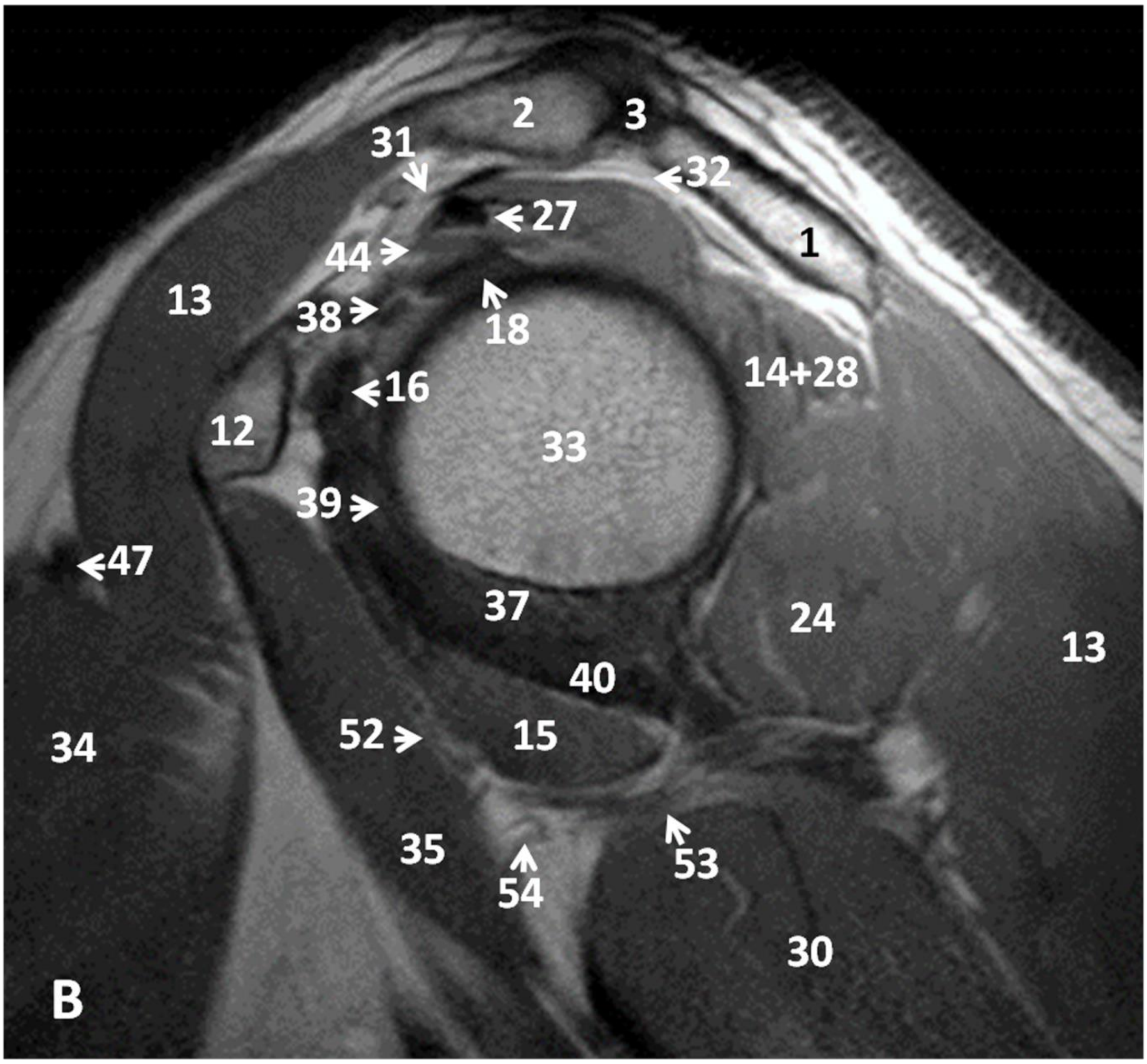
2
3
31
32
27
1
44
13
38
18
16
14+28
12
33
39
47
37
24
13
40
52
15
34
35
54
53
30
B

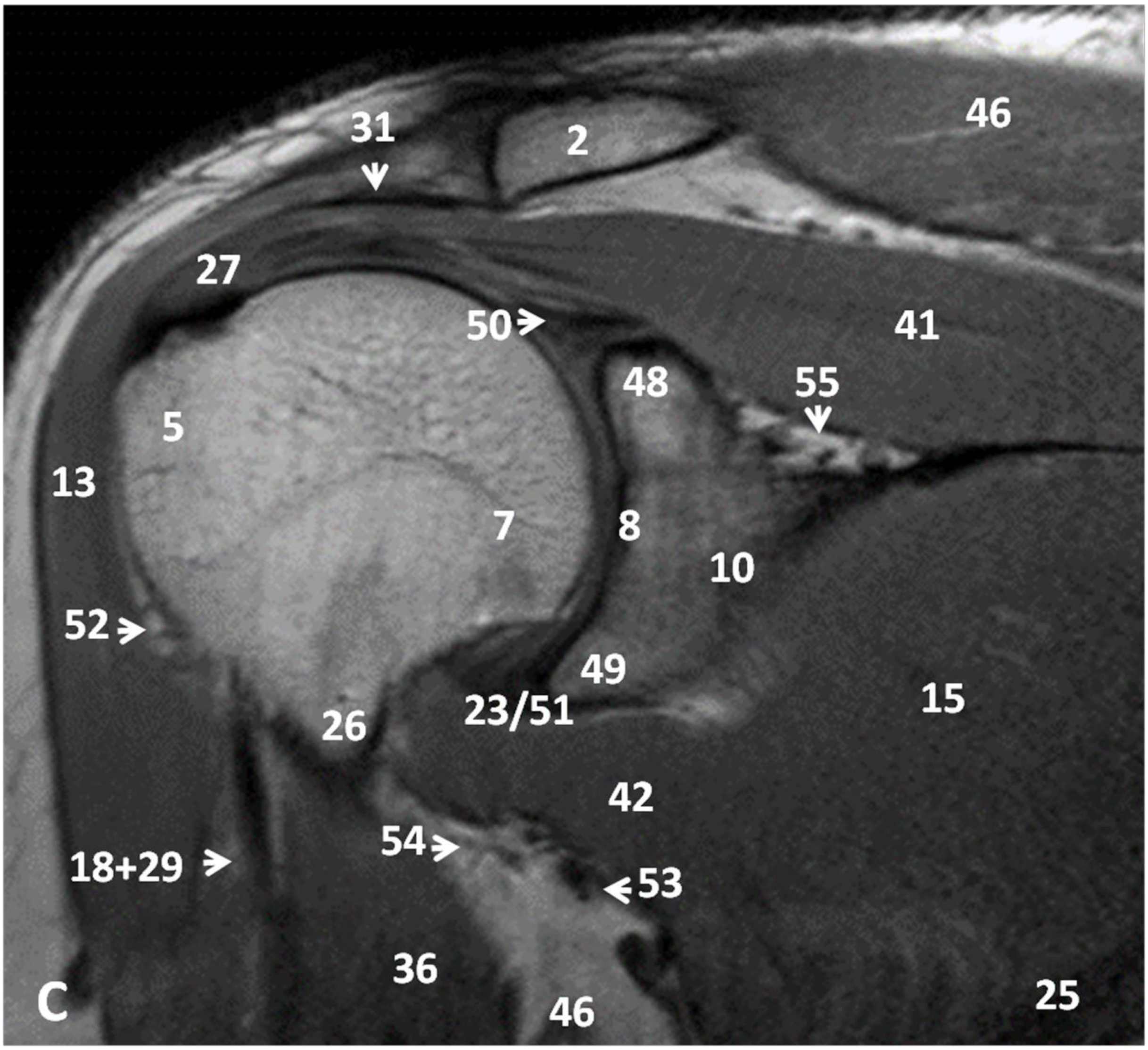

Figure 3, additional material: (A) axial, (B) sagittal oblique and (C) coronal oblique MR anatomy of the shoulder. PD-weighted sections obtained with 3 Tesla device.

1: Acromion, 2: Clavicle, 3: Acromioclavicular joint, 4: Lesser tuberosity, 5: Greater tuberosity, 6: Bicipital groove, 7: Anatomical neck, 8: Glenoid fossa/glenohumeral joint, 9: Scapula, 10: Scapular neck, 11: Suprascapular notch, 12: Coracoid process, 13: Deltoid, 14: Infraspinatus muscle, 15: Subscapularis muscle, 16: Subscapularis tendon, 17: Teres minor tendon, 18: Long head of biceps tendon, 19: Anterior labrum, 20: Posterior labrum, 21: Middle glenohumeral ligament, 22: Suprascapular nerve, 23: Inferior glenohumeral ligament/capsule, 24: Teres minor muscle, 25: Rib, 26: Humeral diaphysis, 27: Supraspinatus tendon, 28: Infraspinatus tendon, 29: Long head of biceps muscle, 30: Lateral head of triceps muscle, 31: Coracoacromial ligament, 32: Subacromial bursa, 33: Humeral head, 34: Pectoralis major muscle, 35: Coracobrachialis muscle, 36: Triceps muscle, 37: Labrum, 38: Superior glenohumeral ligament, 39: Middle glenohumeral ligament, 40: Inferior glenohumeral ligament/capsule, 41: Supraspinatus muscle, 42: Teres major muscle, 43: Pectoralis minor muscle, 44: Coracohumeral ligament, 45: Musculocutaneous nerve, 46: Terminal branches of brachial plexus, 47: Cephalic vein, 48: Supraglenoid tubercle, 49:

Infraglenoid tubercle, 50: Superior labrum, 51: Axillary recess of glenohumeral joint, 52: Anterior circumflex humeral artery, 53: Posterior circumflex humeral artery, 54: Axillary nerve, 55: Suprascapular neurovascular bundle (artery and nerve).

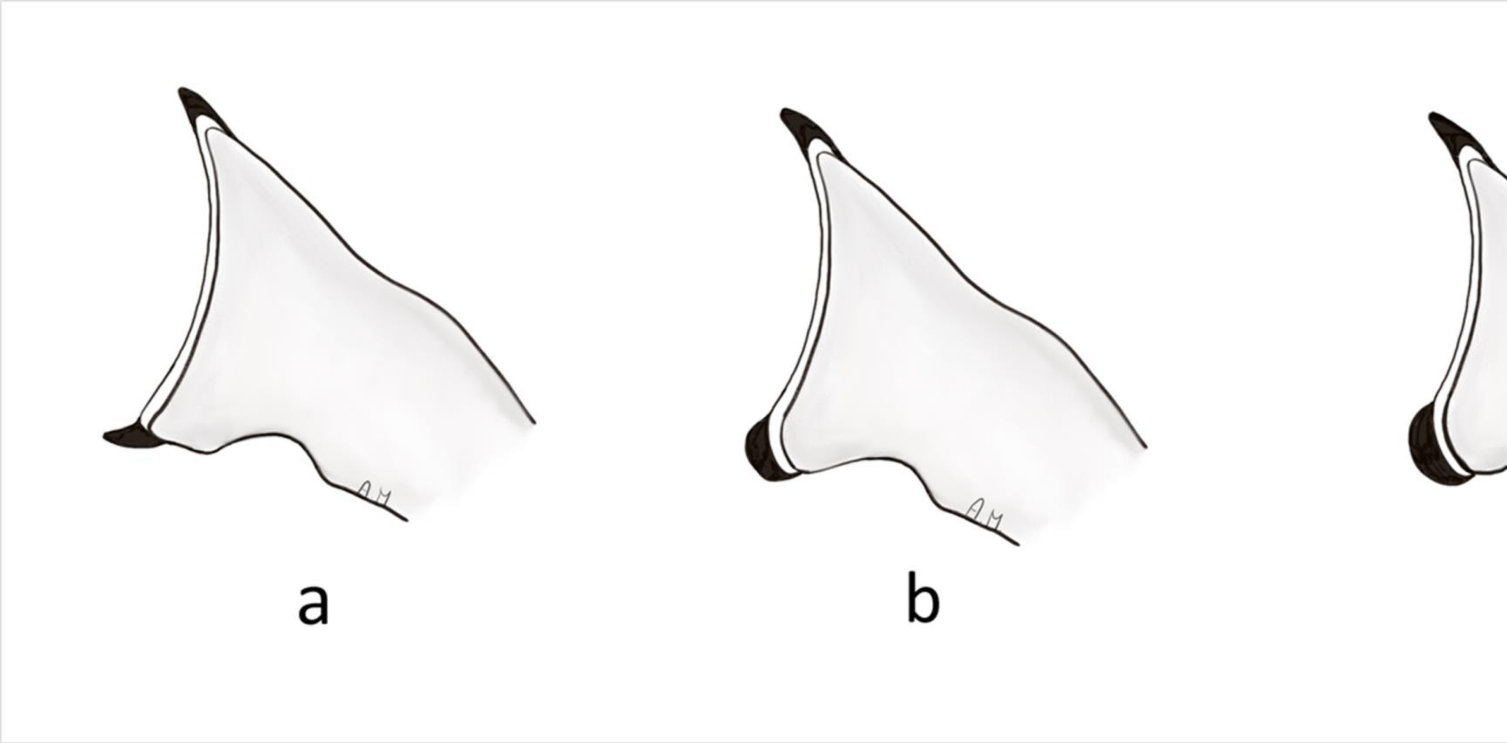

Figure 4, additional material: Schematic illustration of the posterior glenoid rim variants. a: normal triangular; b: rounded or J shaped; c: delta shaped.

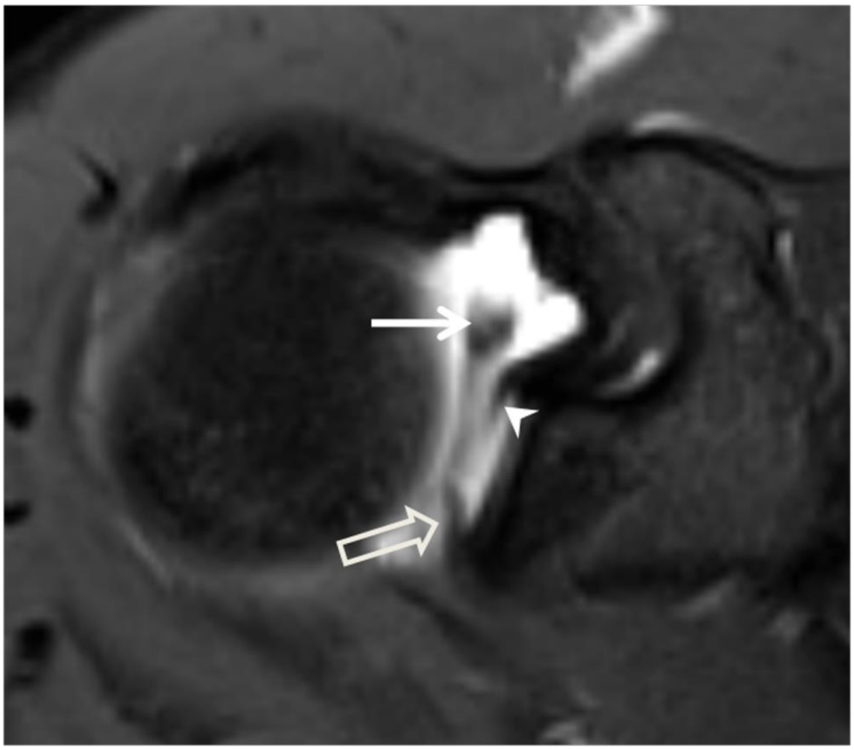

Figure 5, additional material: Biceps labral complex (bicipital anchor). Axial fat-saturated T1-weighted MR arthrographic image shows the common origin (open arrow) of the long head of the biceps tendon (arrow) and of the superior glenohumeral ligament (arrowhead) at the superior aspect of the labrum.

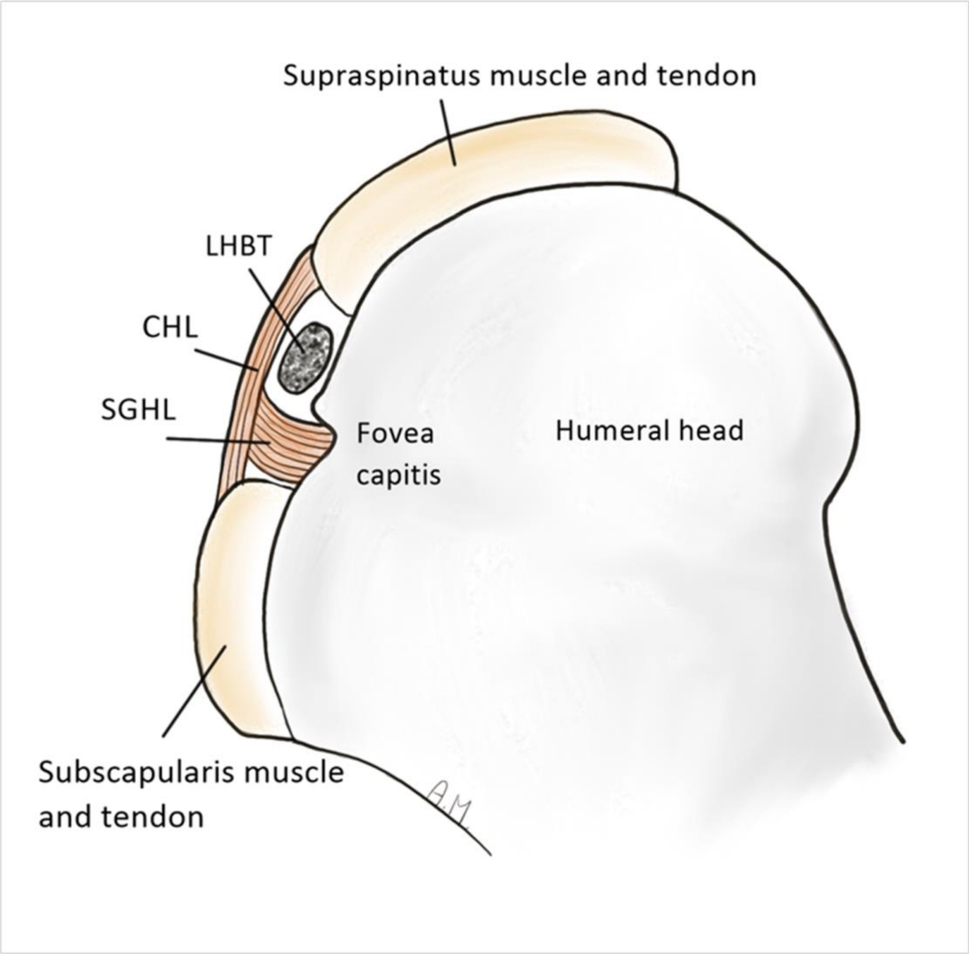

Figure 6, additional material: Schematic illustration of the rotator interval. The rotator interval is located between the supraspinatus muscle and tendon and the subscapularis muscle and tendon and is covered by the coracohumeral ligament (CHL) on a sagittal midsection. LHBT: long head of biceps tendon; SGHL: superior glenohumeral ligament.

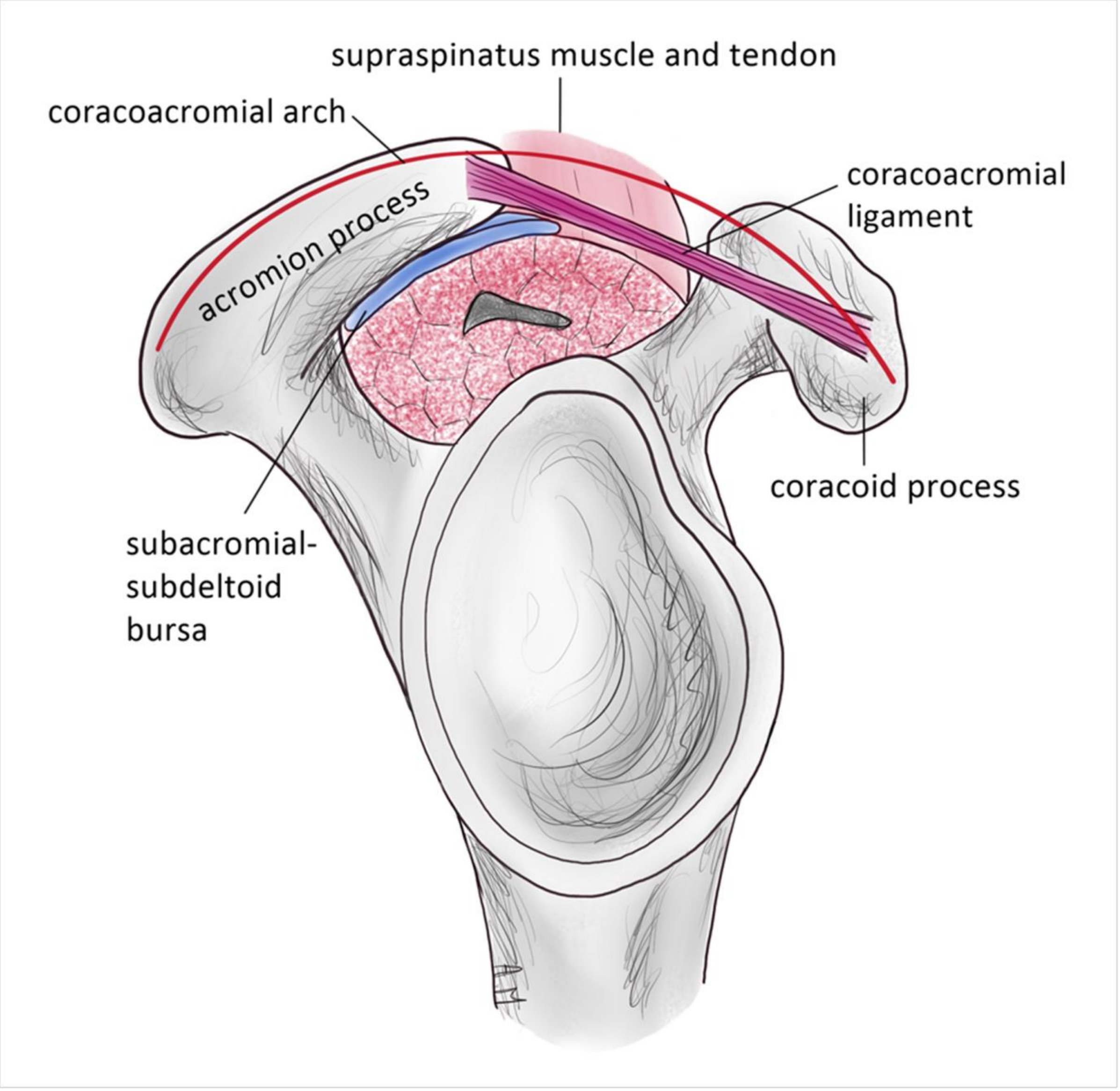

Figure 7, additional material: Schematic illustration of coracoacromial arch (red line) comprising the acromion process, the coracoacromial ligament and the coracoid process. Deep to this arch are the subacromial subdeltoid bursa and the supraspinatus muscle and tendon.

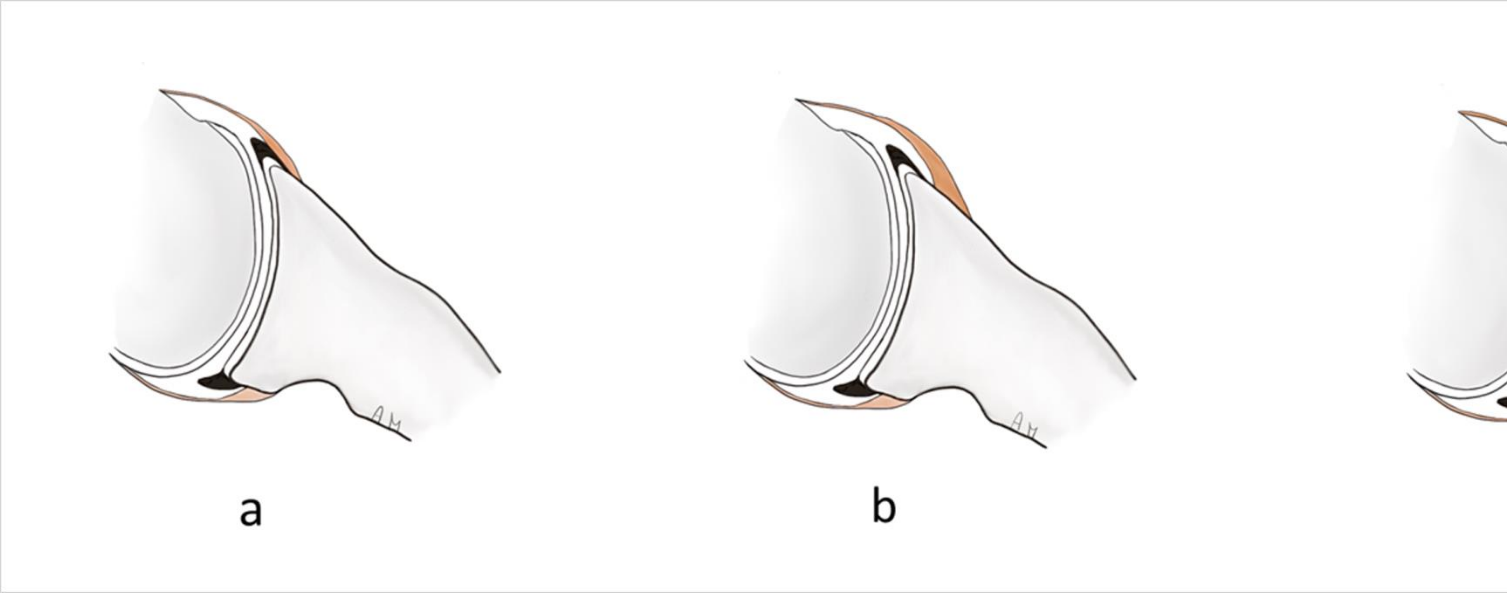

Figure 8, additional material: Schematic illustration of anterior glenoid capsular attachments. a: type I; b: type II and c: type III.

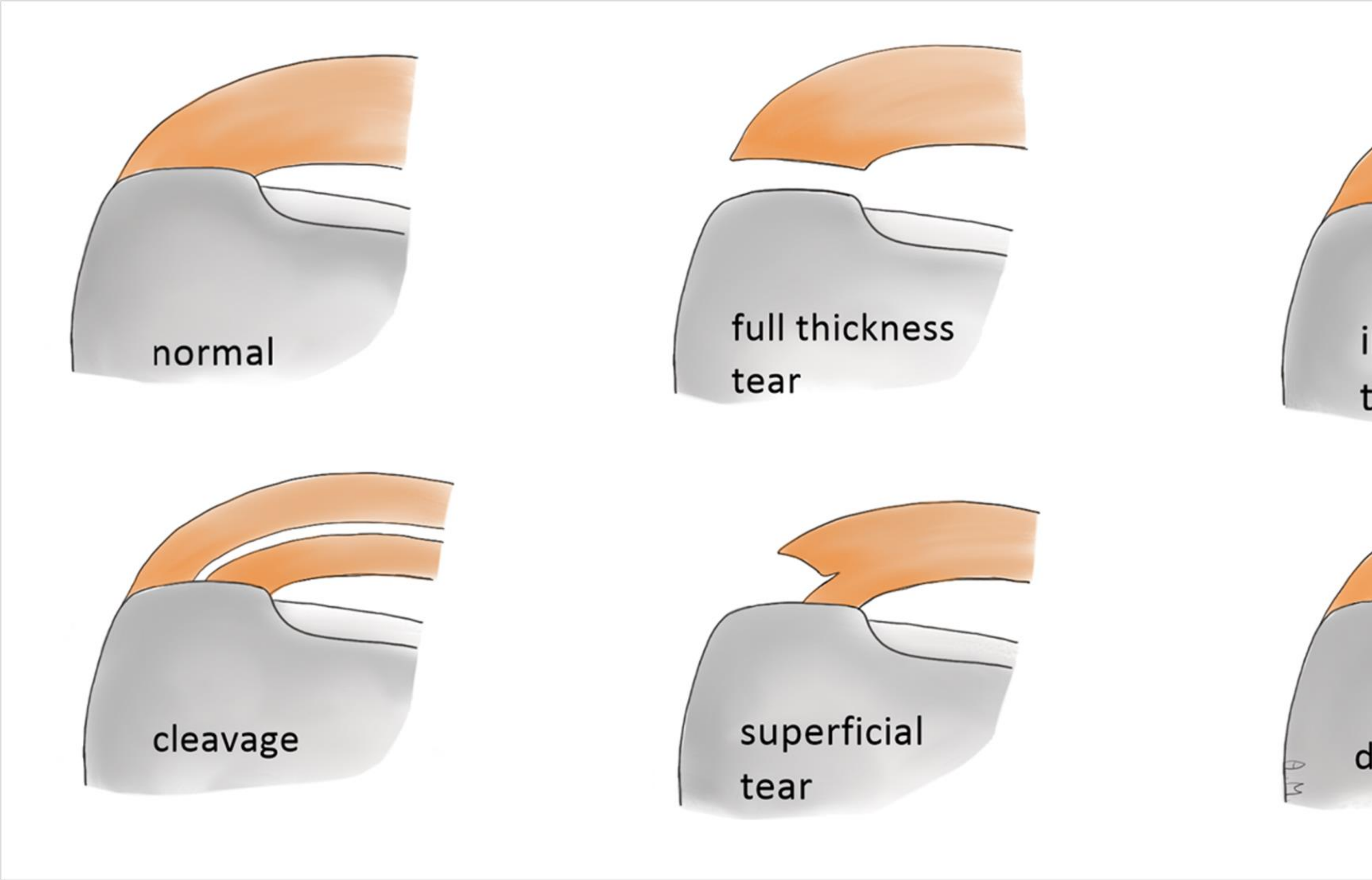

Figure 9, additional material: String theory of the insertion of the supraspinatus tendon. A: The supraspinatus tendon contains two strings representing the superficial and deep tendinous layers. B: Rupture of both strings is a full-thickness tear. C: An interstitial lesion is located between the two strings and limited at the humeral

insertion. D: A cleavage tear is a gap running further between the two strings. E, F: Rupture of one of the two strings is a partial-thickness tear (E: superficial or F: deep tear).

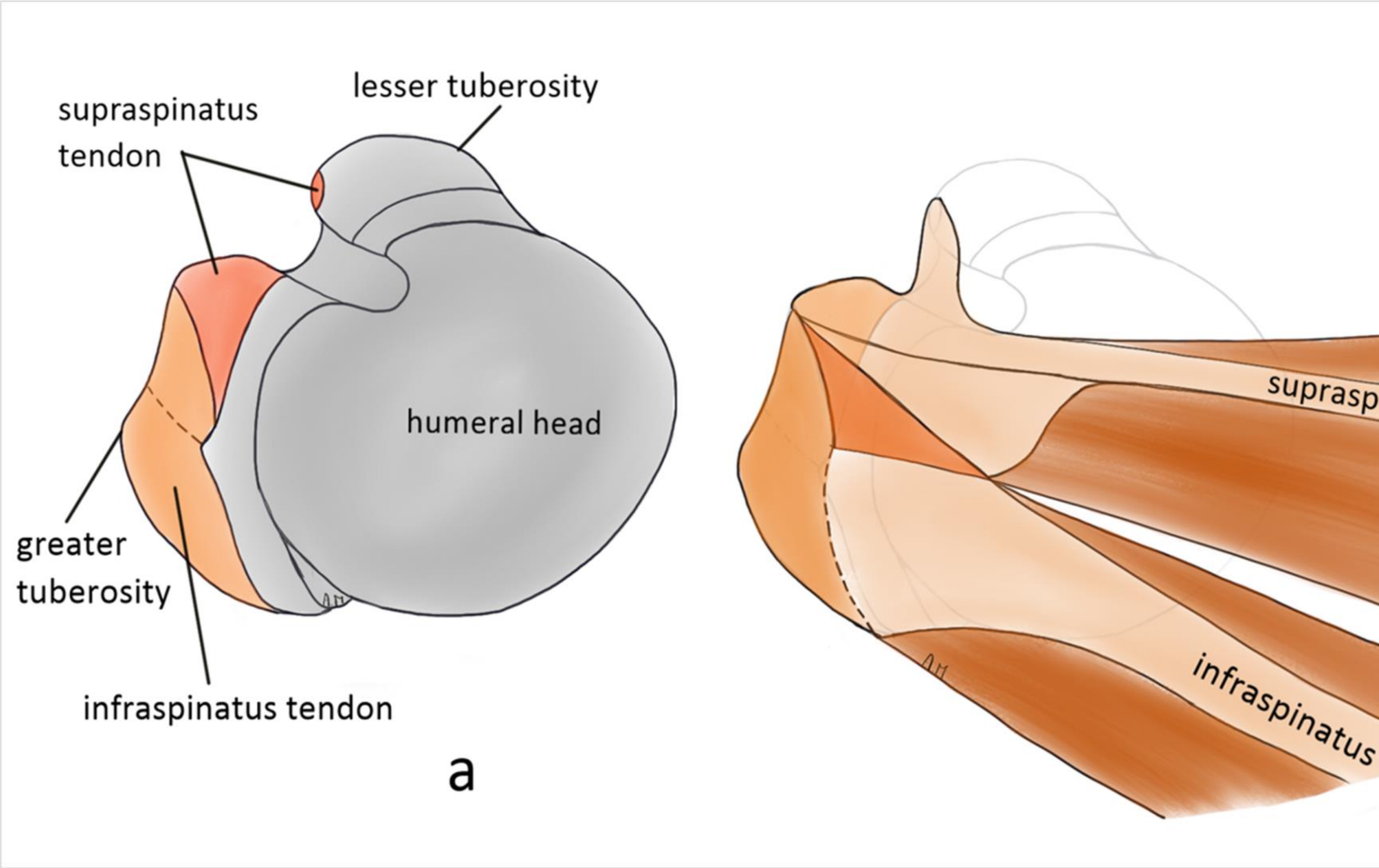

Figure 10, additional material: Schematic illustrations of humeral attachments of the supraspinatus and infraspinatus as viewed from above. The insertion area of the infraspinatus occupies approximately half of the superior facet and the entire middle facet of the greater tuberosity. The insertion area of the supraspinatus is located at the anteromedial region of the superior facet and sometimes at the most superior area of the lesser tuberosity.

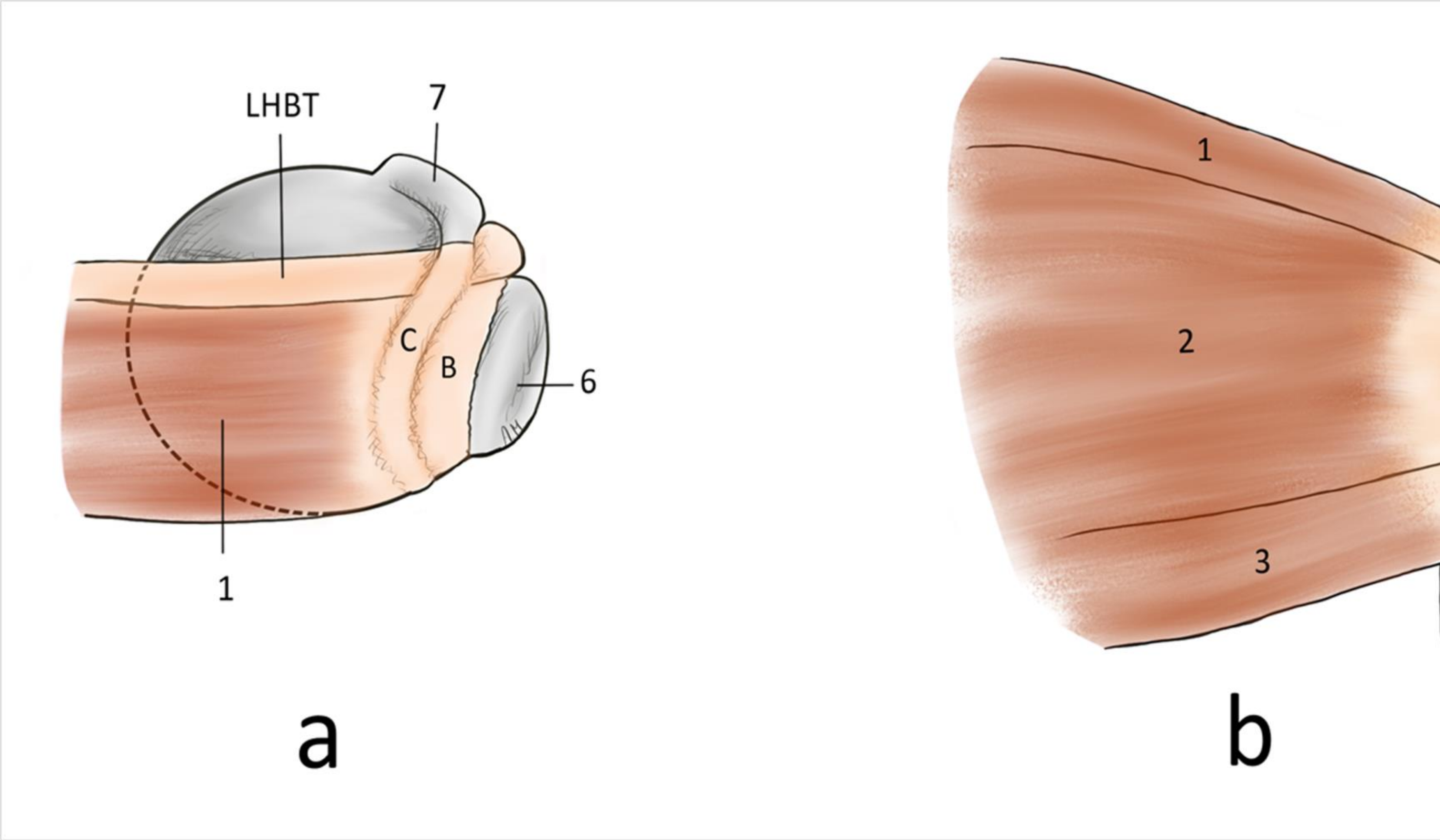

Figure 11, additional material: Rotator cable and crescent. (a) superior view, (b) posterior view. The rotator cable spans to the length of the supraspinatus (1), infraspinatus (2) and teres minor (3) insertions. 4: rotator crescent; 5: rotator cable; 6: greater tuberosity; 7: coracoid process; LHBT: long head of biceps tendon.

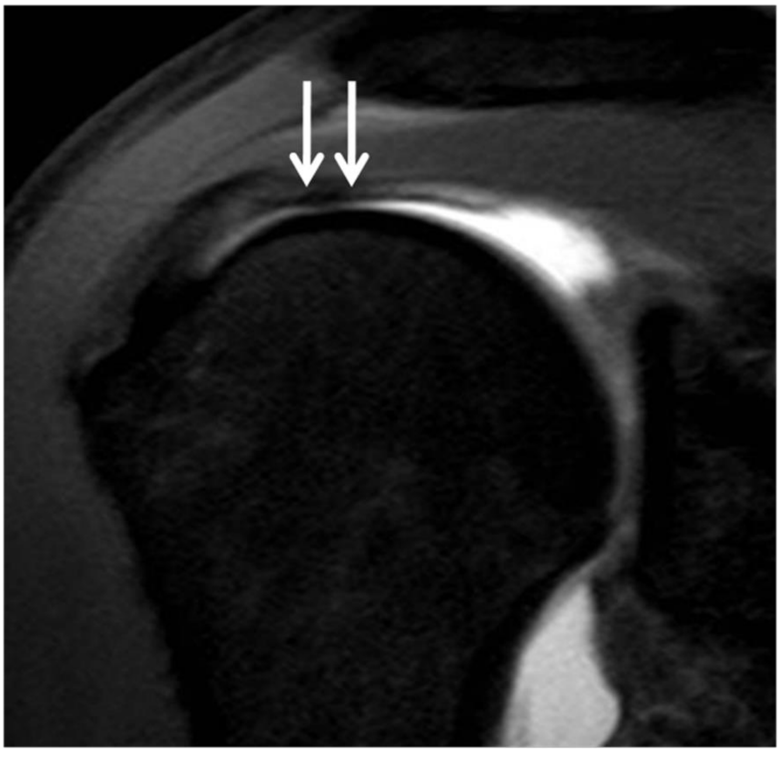

Figure 12, additional material: Rotator cable. Coronal fat-saturated T1-weighted MR arthrographic section shows the rotator cable as a thin hypointense transverse band (arrows) along the articular surface of the supraspinatus tendon interposed between the tendon and the articular humeral cartilage.

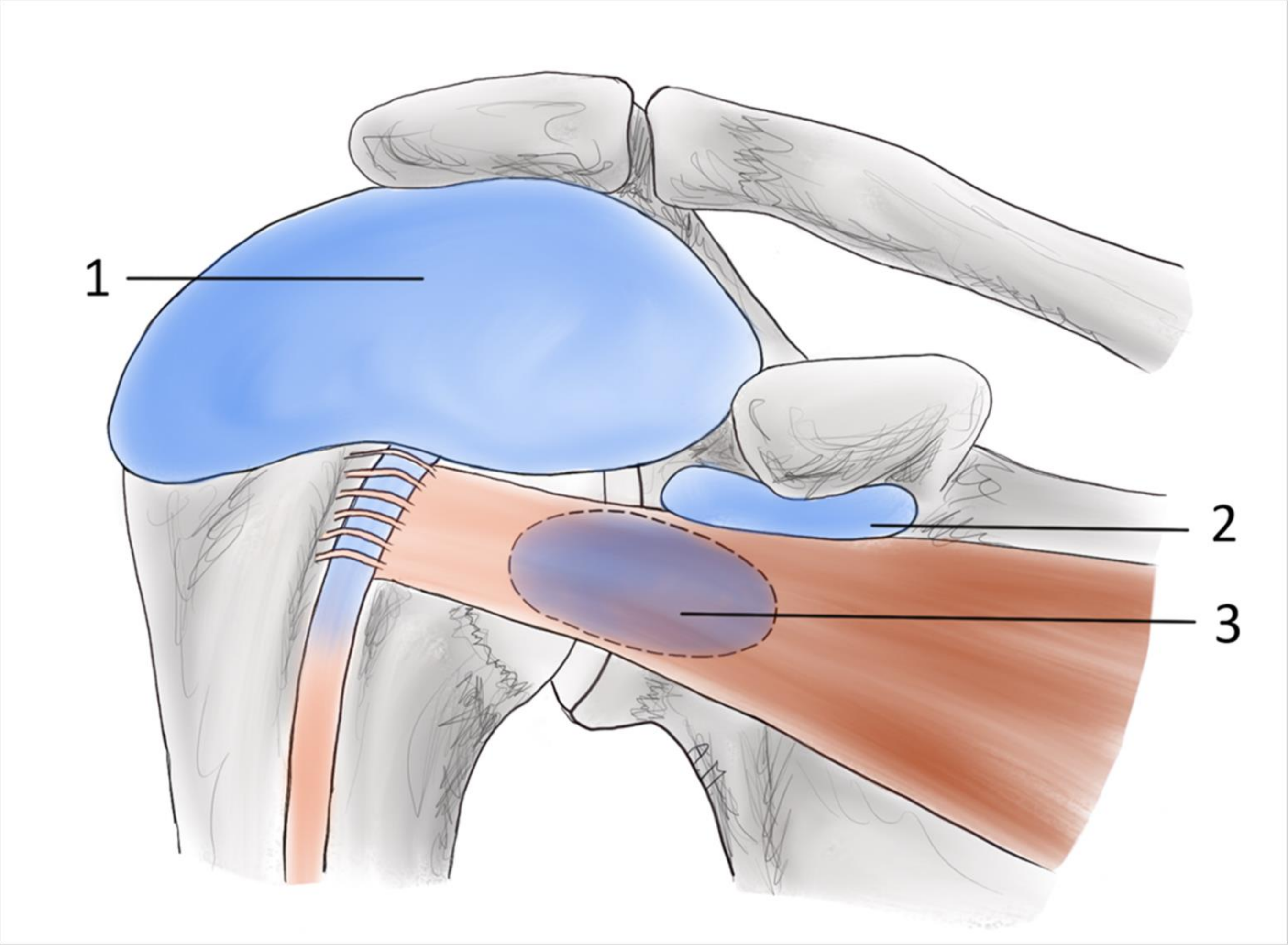

Figure 13, additional material: Most important bursae of the shoulder. 1: subacromial subdeltoid bursa; 2: subcoracoid bursa; 3: subscapular bursa (subscapular recess).

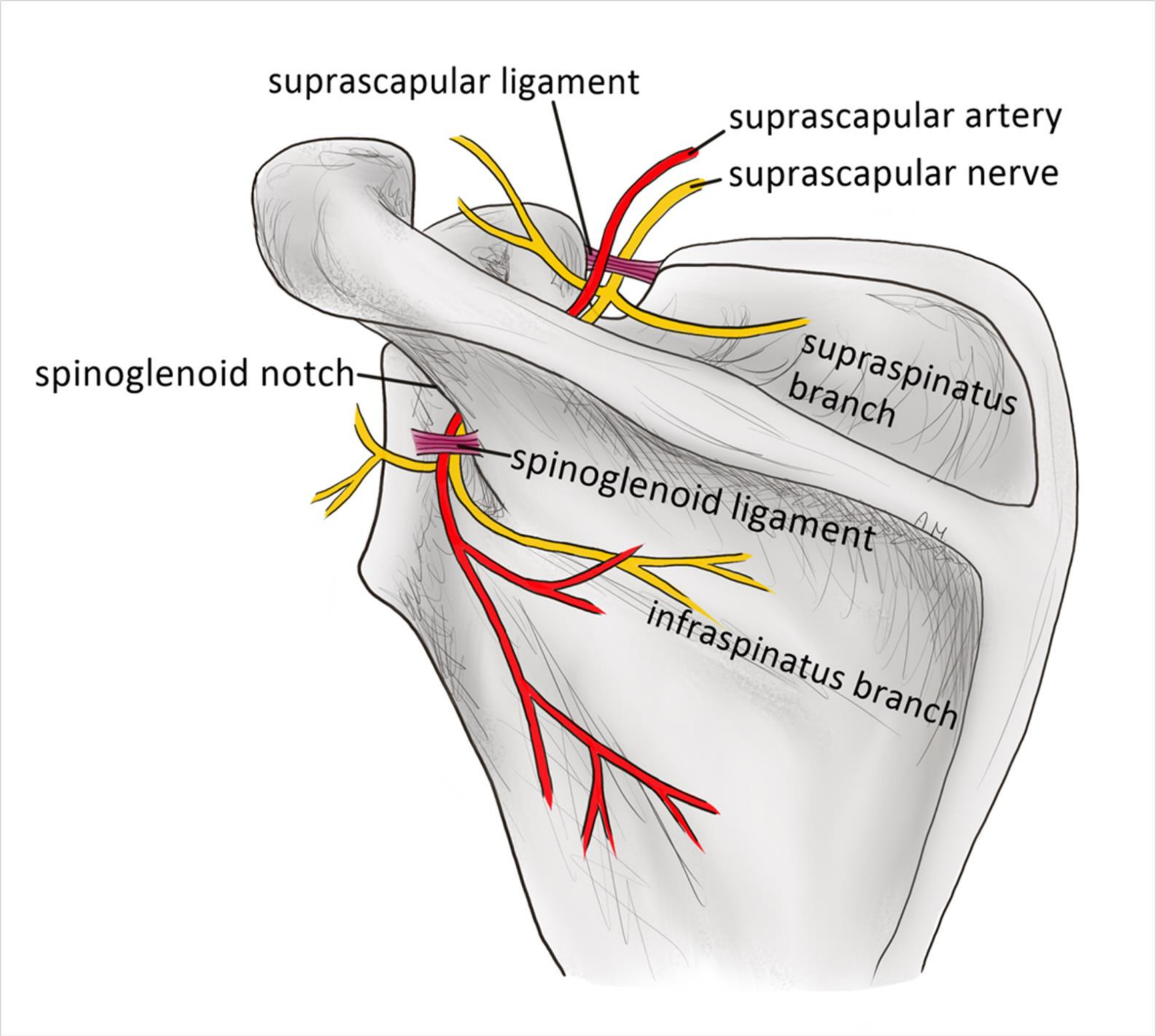

Figure 14, additional material: Suprascapular nerve and branches. The suprascapular nerve traverses posteriorly the suprascapular fossa through the suprascapular notch, passing beneath the suprascapular ligament and sending off several branches. The nerve then traverses the spinoglenoid notch to enter the infraspinatus fossa. The spinoglenoid ligament forms the roof of the notch. Distal to the spinoglenoid notch, the suprascapular nerve divides in two or more muscular branches. The suprascapular vessels (in red) project superior to the suprascapular ligament.
